# Neural and Behavioral Correlates of Pure Tone and Narrowband Noise Processing in Rats: A Tradeoff between Discrimination and Sensitivity

**DOI:** 10.1523/ENEURO.0347-25.2026

**Published:** 2026-04-08

**Authors:** Riccardo Caramellino, Davide Maggioni, Pilar Vaca Sánchez, Michael Harvey, Gregor Rainer

**Affiliations:** Section of Medicine, University of Fribourg, Fribourg CH-1700, Switzerland

**Keywords:** auditory stimuli, auditory thalamus, behavioral detection, neural coding, rodents, sensory disorders

## Abstract

Accurate characterization of auditory pathway responses is critical for understanding neural disorders that affect hearing, including both peripheral and central deficits, as well as broader neurodevelopmental conditions. Animal models provide a way to investigate auditory circuit activation with high spatial and temporal resolution. Here, we first assessed behavioral detection of pure tones (PT) and narrowband noise (NBN) stimuli in male rats, revealing that NBN targets were detected at lower amplitudes than PT targets, consistent with human auditory detection patterns. We then compared these behavioral results with neural responses recorded in female rats’ medial geniculate body (MGB), a thalamic relay to the auditory cortex. Using high-density multichannel recordings, we found that NBN stimuli elicited greater neural sensitivity at low amplitudes, whereas PT stimuli evoked faster responses and higher peak firing rates. MGB units achieved maximal frequency discrimination at amplitudes close to detection threshold, with a sharp decline at both lower and higher intensities, suggesting that neurons with lower sensitivities primarily contribute to processing at moderate to high sound levels. Decoding analyses demonstrated accurate frequency classification from MGB population activity, with PT yielding higher accuracy than NBN, highlighting the potential of multichannel recordings for precise auditory information assessment. Our results indicate that NBN and PT stimuli differentially probe auditory thalamic processing, with NBN advantageous for detecting subtle sensitivity changes and PT optimal for timing analyses. These findings advance the translational relevance of rodent models for preclinical evaluation of therapies targeting central auditory deficits.

## Significance Statement

While pure tones and narrowband noise are both used in humans and animals to study auditory function, the sensitivity at the neural circuit and behavioral level for these two parametric stimulus sets has not been systematically characterized. Here we examine behavioral and thalamic neural responses in the medial geniculate body of the rat to these stimulus sets, revealing overall similarity but also divergences in terms of latency, sensitivity, responsivity, and classification performance. We conclude that stimulus choice critically shapes neural and perceptual sensitivity, showing that specific stimuli are better suited to quantify distinct aspects of auditory function. Our findings support the development of robust preclinical protocols for evaluating auditory dysfunction and enhancing the translational relevance of animal models.

## Introduction

To develop effective interventions for neural disorders affecting auditory processing, it is essential to precisely characterize neural circuit activation along the auditory pathway. Beyond disorders that exclusively target the auditory system in the conductive, sensorineural (Michels et al., 2019) as well as the central nervous system domain (Phillips et al., 2002; Weihing et al., 2015), broader neurodevelopmental or cognitive disorders can also significantly impact auditory processing. For example, individuals with autism spectrum disorder as well as attention deficit hyperactivity disorder exhibit aberrant auditory-evoked potentials (AEP) to tone stimuli, affecting both AEP early negative N and later P3 waves (Oades et al., 1988; Tsai et al., 2012). Similarly, individuals with Down syndrome exhibit reduced hearing sensitivity (Marcell et al., 1995; Huber et al., 2004) and delayed AEP latency (Pekkonen et al., 2007). Whereas P3 waves tend to be associated with cognitive interpretation and significance of the auditory stimuli, the N waves are thought to reflect auditory stimulus characteristics such as frequency, amplitude, or relative rarity. Precise characterization of auditory deficits across disorders is important for diagnosis as well as assessment of potential therapeutic approaches. In this context, preclinical animal studies offer the possibility for detailed characterization of auditory pathway responses using high-density probes in conjunction with sophisticated behavioral paradigms. This work can not only provide overall response profiles in terms of mass action signals, i.e., cortical or brainstem AEP, but also allow characterization of information transfer by neurons at particular stages of the ascending auditory pathway. Progress in validation of animal models along these lines can improve the reliability of preclinical testing of novel therapies, in the context of sensorineural or central neural auditory deficits (Lye et al., 2023).

The choice of auditory stimuli is an important aspect for both establishing a baseline in unimpaired individuals and subsequently characterizing functional deficits in particular disorders. Here, it is crucial to use parametric stimuli that assess auditory responses in a frequency- and amplitude-specific manner. Standardized stimuli such as pure tones (PT) and narrowband noise (NBN) appear particularly suitable for this purpose, as they provide precise spectral control, unlike natural sounds, chords, or chirps that encompass broad and structured frequency content (Theunissen and Elie, 2014; Angeloni and Geffen, 2018). Indeed, it has been suggested that developing standardized protocols would be highly desirable for preclinical assessments (Le Prell, 2023). A pertinent issue in this context is whether PT and NBN provide equivalent information about auditory stimuli or whether there are particular characteristics that distinguish these two parametric stimulus sets in terms of revealing information transmission in the auditory pathway. Generally, studies of auditory pathway activity continue to employ PT stimuli to systematically assess amplitude- and frequency-dependent neural activations and how these are affected by various experimental manipulations (Anderson et al., 2009; Bäuerle et al., 2011; Christensen et al., 2019; Ishizu et al., 2021; van Zwieten et al., 2021). NBN stimuli have in contrast been rarely used for this purpose, but notably a study in marmoset monkeys has performed extracellular recordings in the primary auditory cortex and caudomedial belt area, showing that NBN evoked stronger responses with lower thresholds and shorter latencies than PT, while largely yielding similar frequency tuning (Kajikawa et al., 2011). This suggests that NBN might be preferable to PT for detecting small to moderate aberrations in auditory pathway activations that may occur during ageing or in conjunction with various disorders which impact hearing. Given the availability of rodent models for various disorders, we set out here to examine differences in neural auditory circuit activation in the rat to PT and NBN stimulus sets. This comparison is useful to determine the advantages or disadvantages of each stimulus class when exploring particular features of sensorineural or central alterations. We focus on neural activations within the medial geniculate body (MGB), the principal thalamic relay nucleus that conveys auditory information from brainstem nuclei to the cerebral cortex (Winer, 1984). We selected the MGB because there is considerable homology in the connectivity of MGB between humans and rodents (Keifer et al., 2015), suggesting that findings in rodents may also be relevant for human auditory processing. Furthermore, MGB activity has been linked to disease-related auditory dysfunctions, for example, in the context of neurodevelopmental disorders (Anderson and Linden, 2016) as well as tinnitus (Caspary and Llano, 2017). Studying the MGB provides an informative window on bottom-up auditory signal transmission, as it represents the major input to auditory cortical circuits involved in auditory perception and decision-making.

We performed high-density multichannel probe recordings in the MGB, presenting PT and NBN stimulus sets carefully matched for frequency, intensity, and duration in Sprague Dawley wild-type rats. Based on available results in the cortex (Kajikawa et al., 2011), we anticipated higher sensitivity and shorter latencies of MGB units for NBN compared with PT stimuli. In parallel to studying neural circuit MGB activations, we also investigated the perceptual ability of rats in an auditory detection task, where subjects made lever responses to randomly interleaved PT and NBN targets presented at different amplitudes. This approach allowed us to examine differences between the two stimulus sets both in terms of neuronal activation and behavioral performance.

## Materials and Methods

All experimental procedures were approved by the Canton of Fribourg committee on animal experimentation. The study was funded by SNF Project 212255 and Synapsis Foundation Project 2022-PI07.

### Subjects

Behavioral testing was conducted on eight adult male Sprague Dawley rats, aged 7–8 months, with an average body weight of 580 ± 60 g. Neurophysiological data were collected from four adult Sprague Dawley female rats, aged between 4 and 7 months and weighing on average 360 ± 30 g. Rats were bred and housed in our animal facility under controlled environmental conditions and a light/dark cycle with gradual transitions (light phase midnight–noon). Experiments described here were performed in the second half of the light phase, generally between 8 A.M. and noon. Animals participating in the behavioral protocol received controlled food access during training and testing phases and continuous access to water.

### Behavioral task and training

Behavioral sessions were conducted in a custom-built sound-attenuated operant chamber (32 cm × 32 cm × 55 cm) equipped with a lever, a house light, and a reward light positioned at the food trough. A speaker and a camera were mounted 50 cm above the chamber to deliver auditory stimuli and monitor behavior, respectively. The speaker was positioned facing downwards at the center of the chamber ceiling. As the animals typically pressed down the lever facing the wall behind the lever, they were well within range of the speaker and in a defined location during sound presentation in this otherwise unconstrained behavioral paradigm. The entire setup was controlled via a PC using custom MATLAB scripts. The acoustic background noise level in the behavioral chamber was 27 ± 2 dB sound pressure level (SPL). The target stimuli used in the behavioral task were analogous to those employed in the electrophysiological experiments (see below, Auditory stimulation and recordings). Initial training employed a 7–10 kHz NBN target. Training was divided into four phases. Phase 1: We manually shaped animals’ behavior to teach them to press a lever in order to receive a food reward (45 mg chocolate-flavored pellets; TestDiet), which was paired with the presentation of the auditory target (100 ms duration at 60 dB SPL). Phase 2: Animals were trained to press and hold the lever until the target was presented. The target was initially delivered 20 ms after lever press, and this delay was incrementally increased by 2 ms following each correct trial, defined as releasing the lever within 700 ms of stimulus onset. Incorrect responses (missed trials) resulted in a 5 s timeout, during which all lights were turned off. Once all animals reliably responded at a delay of 2.5 s, Phase 3 began, where the target was presented at a random time point between 1 and 3.5 s after lever press. Training continued at this stage until animals achieved a performance criterion of ≥90% correct responses. In Phase 4, the target stimulus was presented at one of eight amplitudes (28, 31.5, 35, 38.5, 42.5, 46, 49.5, or 53 dB SPL) with equal probability, and in one of two forms: a PT (8.5 kHz) or NBN centered at 8.5 kHz (7–10 kHz, 1/2-octave bandwidth). Targets were presented in a pseudorandomized fashion, with target onset times varying randomly between 1 and 3.5 s. Animals were rewarded for correctly releasing the lever within 700 ms of stimulus onset and received a 5 s timeout for incorrect responses. Behavioral performance and stimulus–response data were recorded using custom MATLAB scripts.

### Surgical procedure

For acute extracellular recordings, anesthesia was induced by an intraperitoneal injection of ketamine (Ketasol, 100 mg/kg) and xylazine (Xylazol, 20 mg/kg) and maintained throughout the procedure with inhaled isoflurane (∼0.5–3.5%) in pure oxygen. Body temperature was maintained at 37.5°C using a homeostatic heating pad (Kent Scientific). We applied eye ointment during the procedure to protect the animal's eyes. Rats were positioned in a stereotaxic frame, and, following a localized lidocaine injection (0.5 ml of 1% lidocaine), a midline incision was made on the scalp to expose the skull. The skin and the muscle were retracted to expose the left side of the skull above the auditory cortex. A craniotomy (3 mm × 3 mm) was performed over the target area, and we carefully made a slit in the dura to allow electrode insertion. Craniotomy coordinates were established based on the rat brain atlas (Paxinos and Watson, 2006) and vascular landmarks, allowing for probe insertion into the auditory thalamus at a 60° angle relative to the brain surface.

Extracellular recordings were acquired from the medial geniculate body (MGB) under isoflurane anesthesia (∼0.5–3.5%, generally 1–1.5% during data acquisition) using a high-density single-shank Neuropixel 1.0 probe (Imec). Probes were inserted at anteroposterior (AP) positions ranging from −5 to −6.5 mm relative to bregma and dorsoventral positions from 3 to 4 mm. Insertion depth was optimized in each case by gradually lowering the probe while monitoring real-time neural activity (Open Ephys GUI) until auditory-evoked responses were detected (∼4–5 mm depth from the brain surface). Functional mapping and postmortem histological reconstructions were used to confirm accurate targeting of the MGB.

### Auditory stimulation and recordings

Anesthetized rats were placed inside a sound-attenuated chamber and presented with auditory stimuli delivered via an electromagnetic speaker (TDT) positioned in front of the animal at 25 cm distance from the nose. The background noise level in the recording setup was measured at ∼31 dB SPL, primarily originating from surrounding equipment such as the computers and the anesthesia machine. Stimulus delivery was controlled using custom MATLAB code interfaced with an RZ6 multi-I/O processor (TDT). Animals were presented with an equal number of pure tone (PT, sine wave) and narrowband noise stimuli (NBN), matched in amplitude, frequency, and duration. Stimuli covered 11 frequency bands spaced by half octaves (from 500 to 22,000 Hz) and eight sound pressure levels (40–75 dB SPL, in 5 dB increments). Sound pressure level (SPL) measurements were performed using a calibrated miniDSP UMIK-1 measuring microphone positioned at the animal's head location. All SPL values reported in the manuscript refer to RMS measurements. Acoustic stimuli were calibrated using a procedure that mapped speaker driving voltages to corresponding sound pressure levels (SPL). This calibration was performed separately for PT and NBN, for both the terminal recording and behavioral setups. The accuracy of the calibration was verified by multiple SPL measurements during the experiments using the same procedure to verify their stability across the experimental schedule. NBN stimuli were generated by applying a second-order Butterworth bandpass filter to white noise; they had a 1/2-octave bandwidth and were centered on the same frequencies as the corresponding PT stimuli. The stimulus set included the following 1/2-octave-spaced center frequencies (base-2 spacing): 560, 800, 1,120, 1,600, 2,240, 3,150, 4,500, 6,300, 9,000, 12,500, and 18,000 Hz, with NBN bandwidths defined by the corresponding 1/2-octave boundaries to ensure full coverage of the range without overlap. Individual stimuli lasted 100 ms and were followed by a randomized intertrial interval between 200 and 300 ms. A 10 ms cosine amplitude ramp was applied at stimulus onset and offset to minimize nonspecific onset responses. Presentation blocks contained 15 repetitions (trials) of each stimulus condition. Neural activity was digitized at a sampling rate of 30 kHz through the Open Ephys GUI's Neuropixel PXI plugin on the first 384 recording channels from the probe tip, with external referencing (ground and reference connected via a wire bridge). Stimulus onset times were digitalized and acquired via the Open Ephys GUI's NI-DAQmx plugin. Synchronization between neural data and stimulus timestamps was achieved by sending a shared 1 Hz digital square wave signal to both acquisition systems. All data were stored on a PC for offline analysis. Spike sorting was performed offline using the Kilosort3 package (Pachitariu et al., 2023) to isolate single and multiunits. Following automated spike detection and clustering, data exploration was performed using the Phy Template GUI (https://github.com/cortex-lab/phy) to evaluate data integrity. Manual curation after the automatic clustering was avoided to reduce the risk of introducing experimenter bias. We used a total of 895 MGB units in the subsequent analyses.

### Histology

At the end of the surgery, the animal was deeply anesthetized with an overdose of pentobarbital (200 mg/kg, i.p.) and perfused transcardially with 400 ml of phosphate-buffered saline, followed by 400 ml of 4% paraformaldehyde. The brain was fixed overnight in 4% paraformaldehyde and subsequently cryoprotected in 15% and then 30% sucrose solution until the tissue sank. Serial coronal sections (40 μm) were cut using a sliding microtome (Leica) and Nissl stained for verification of the electrode track. A DiI-coated electrode was used to validate the electrode insertion coordinates.

### Statistical and decoding analyses

#### Detection theory

To apply a signal detection theory framework to our behavioral data, we segmented each trial into four temporal components following a method previously described (Wang et al., 2023). We divided the trials into two segments based on target onset time, each lasting 1,250 ms. For trials where the target appeared between 1,000 and 2,250 ms, any response that occurred after target onset but within the 700 ms response window was classified as a hit. Responses made after this window were considered misses. For the second segment (onset times from 2,250 to 3,500 ms), we defined a response window identical to the hit window and used it to identify false alarms. From the animal's perspective, the “hit” and “false alarm” (FA) windows were indistinguishable in timing—the only difference was that no target was presented during the “false alarm” window. If the animal withheld a response during this period, the trial was counted as a “correct rejection.” Importantly, this classification scheme was applied only during analysis; in practice, animals were rewarded exclusively for lever release following stimulus presentation. Any responses occurring before the “hit” (and by extension “false alarm”) windows were labeled as aborted trials and not considered in the signal detection analyses. Based on these definitions, we computed the following:

$$\mathrm{Performance}\;=\frac{\mathrm{HT}+\mathrm{CR}}{\mathrm{HT}+\mathrm{CR}+\mathrm{FA}+\mathrm{MS}},$$

$$\mathrm{Behavioral}\;\mathrm{sensitivity}\;(d^{\prime })=Z(\mathrm{HT})-\;Z(\mathrm{FA}),$$

where *Z*(HT) and *Z*(FA) are the *z*-transformed hit rate (HR) and false alarm rate (FAR), respectively.

#### Responsive units detection

To identify auditory-responsive neurons, we performed a two-way repeated measures ANOVA with factors frequency and amplitude on the spike rate in a response window of 5–95 ms post target sound onset across trials (*n* = 15) separately for PT and NBN stimuli. A unit was considered responsive, if for at least one frequency there was a significant effect of amplitude on the neural response (post hoc multiple comparison with Bonferroni’s correction, *p* < 0.01).

#### Frequency response area and log-normal fitting

For neurons identified as responsive, we computed the frequency response area (FRA) by averaging firing rates across all trials for each stimulus condition. To characterize the sensitivity of each neuron to sound intensity at individual frequencies, we fit the neural responses across amplitudes at each frequency using a log-normal (LN) function of the form:

$$f(x)=\frac{E}{x\cdot \sigma \sqrt{2\;\pi }}\cdot e^{-\frac{{\left(\log\;(x)-\mu \right)}^{2}}{2\sigma^{2}}}+B,$$

where *x* is the sound pressure level (SPL) and the free parameters *E*, *µ*, *σ*, and *B* represent the response magnitude scaling factor, the log-transformed response center, the dispersion (spread) of the response, and the baseline firing rate (FR), respectively. All free parameters were constrained to be strictly positive to prevent fitting negative firing rates, and upper bounds were set empirically based on the distribution of parameter values in our dataset (upper bounds: [150 20 10 0.4]). We chose to keep B (baseline activity) as a constrained but free parameter rather than fixing it to the measured spontaneous activity, as this yielded substantially more accurate fits across the population. Fits were performed independently at each frequency using nonlinear least-squares fitting. Goodness of fit was estimated using the coefficient of determination (*R*^2^).

#### Estimation of C50 and characteristic frequency

For each log-normal fit between neural response and SPL, we identified the C50 value, defined as the SPL at which the log-normal fit reached 50% of its range between baseline and maximum response. C50 values served as a quantitative measure of neuronal sensitivity to sound intensity at each frequency. Additionally, we estimated the half-width (HW) of each amplitude–response curve as the SPL range over which the response exceeded 50% of its maximum. Neurons displaying nonmonotonic (O-shaped) amplitude–response functions, characterized by an initial rise and subsequent fall in firing rate at higher SPLs, were identified based on the width of the fitted log-normal curve and verified by visual inspection of the FRA. Specifically, units for which the LN fit produced a finite half-width were classified as O-shaped, whereas monotonic units exhibited an effectively infinite half-width. To ensure the robustness of the sensitivity estimates, a set of stringent selection criteria was applied before extracting C50 values: (1) The fit's *R*^2^ for the frequency value was required to exceed 0.7 to guarantee goodness of fit. (2) The maximum firing rate estimated from the LN fit (Rmax) for the frequency was required to surpass 50% of the maximal firing rate observed across all frequencies for that unit. (3) The half-width (HW) of the fitted curve was constrained to be <2 octave bands, ensuring selectivity rather than broadly distributed responses. (4) The *p*_ANOVA_ for the frequency had to be below 0.01. For each unit, the characteristic frequency (CF) was then determined as the frequency corresponding to the lowest C50 value, among those frequencies satisfying these four conditions. The characteristic frequency corresponds to the frequency at which the neuron is most sensitive, with sensitivity being inversely related to the C50 value at CF. A manual curation step was performed to exclude a minimal number of units with atypical response patterns. These instances were identified based on visual inspection of FRAs and LN fits, including instances of spurious fits that passed statistical criteria. This procedure ensured that the population of auditory thalamic units used for all analyses was made of units with a precise frequency tuning.

#### Thalamus tonotopy reconstruction

To reconstruct the tonotopic organization, we analyzed data from each probe penetration across experimental sessions. For each penetration, we selected neurons that exhibited a well-defined characteristic frequency (i.e., those with significantly frequency-selective responses). Using the depth of probe insertion and the known geometry of the probe, we calculated the coordinates of each recorded unit. To localize these neurons anatomically, we identified the corresponding coronal brain section from the rat brain atlas based on the AP coordinate of the penetration. Taking into account the angle of the probe insertion, we superimposed the recording sites onto the atlas image. Each neuron was color-coded according to its characteristic frequency, enabling the visualization of tonotopic gradients across the sampled region. This tonotopic characterization was limited to the lower portion of the rat audiogram given our stimulus range (0.5–22 kHz).

#### Frequency tuning width

To determine the frequency tuning bandwidth, in frequency bands, of each unit, we computed the bandwidth 10 dB above the unit's C50 at its CF. Specifically, we counted the number of frequency bands around the CF for which the C50 was lower than the best C50 + 10 dB, considering only frequencies where the log-normal fit met all criteria described above, Estimation of C50 and characteristic frequency. To assess potential differences at specific amplitudes, we repeated the analysis by computing the bandwidth at each tested amplitude independently of the unit's C50 and compared the resulting distributions of tuning bandwidth.

#### Estimation of time constants

To quantify the temporal persistence, or slowness, of neuronal responses to auditory stimuli, we estimated the time constant *τ* of the exponential decay of interspike interval (ISI) distributions for each recorded unit. Interspike intervals (ISIs) were computed as the temporal differences between consecutive spikes within each trial, considering only ISIs shorter than 30 ms. This threshold was chosen to focus the analysis on the stimulus-driven component of the response, while excluding longer intervals likely due to spontaneous activity. The distribution of ISIs was binned at 1 ms over the 0–30 ms range. Histograms were normalized to obtain probability density functions of ISIs for each unit and stimulus condition. The temporal decay of the ISI distributions was modeled using a single-exponential function of the form:

$$f(t)=A\cdot e^{-\frac{t}{\tau }}+C,$$

where *t* is the interspike interval, *τ* is the time constant of decay (in seconds), and *A* and *C* are free parameters representing the initial response amplitude and baseline offset, respectively. The model was fitted to the normalized ISI histograms using nonlinear least-squares curve fitting (MATLAB's *lsqcurvefit* function) with initial parameter estimates of [*A*, *τ*, *C*] = [1, 1, 1] and lower and upper bounds of [0, 0, 0] and [+∞, 1, +∞], respectively. For each fit, the goodness of fit was quantified using the coefficient of determination (*R*^2^).

#### Analysis of frequency selectivity using signal detection theory (*d*′)

To quantify the frequency selectivity of single units, we applied a signal detection theory-based approach, modeling our data as a two-alternative forced choice task where neural responses are used to discriminate between the unit's characteristic frequency (CF) and an adjacent frequency band. Spike counts were extracted from evoked and baseline windows of 50 ms duration (starting 5 ms after stimulus onset), separately for each unit, protocol, and amplitude of the stimulus. For each trial, we computed the difference in response between the CF and the baseline (ΔCF) and between an adjacent frequency and its baseline (Δadj). Trial-by-trial discriminability was then expressed as the difference between these measures (*D* = ΔCF − Δadj). We defined the probability of correct discrimination (Pc) as follows:

$$\mathrm{Pc}=\frac{n_{D>0}+0.5\;*\;(1+\;n_{D=0})}{n_{\mathrm{total}}+1},$$

where *n_D_*_ > 0_ is the number of trials where *D* > 0, *n_D_*_ = 0_ is the number of trials where *D* = 0, and *n*_total_ is the total number of trials (*n_D_*_ > 0_ + *n_D_*_ < 0_ + *n_D_*_ = 0_). The second term in the numerator assigns half-credit to trials in which the decision variable *D* is exactly zero, i.e., when it provides no information to guide the choice. Cases with *D* = 0 occur when the unit produces identical evoked-baseline differences for the CF and the adjacent frequency (e.g., when no spikes occur on either trial); the term Pc was then converted to *d*′ using the relation *d*′ = √2 × norminv(Pc). When the CF was not located on the border of our frequency range, *d*′ was averaged across the two adjacent frequency comparisons (left and right of the CF); if the CF was at a border, only the available side was used.

#### Decoding analysis

To assess the discriminability of auditory stimuli based on population neural responses, we performed a decoding analysis using multiclass support vector machine (SVM) classifiers. For each recorded unit, spike times were aligned to stimulus onset, and spike counts were computed within a predetermined temporal window. For each trial, spike counts within the selected window were computed separately. For each condition (PT and NBN), neurons, responsive to that condition, were randomly grouped into pseudopopulations of 50 units (see Extended Data Fig. 7-1 for a comparison of decoder performances with different population sizes). To improve the reliability and generalizability of the results, 10 pseudopopulations were generated for each condition. These populations were constructed by random selection of units from the full responsive pool for each condition (PT, 699 units; NBN, 653 units), with selection allowed to occur with replacement, although the large pool sizes minimized actual overlap across the 10 populations of 50 units. Selection was performed without any constraint based on unit properties such as CF or C50, to ensure an unbiased sampling and to prevent oversampling of specific parameter ranges. The analysis was not limited to populations of simultaneously recorded units from a single penetration, as this constraint would have resulted in insufficient coverage of the stimulus frequency range. Instead, our approach characterized population-level response properties across a group of animals and, separately, within single recording sessions encompassing multiple penetrations (see Extended Data).

Decoding was performed using a multiclass SVM implemented via MATLAB's *fitcecoc* function classifier (linear kernel, box constraint optimized using Bayesian optimization), which uses a one-versus-one error-correcting output codes strategy to handle multiclass problems. The classifier input was a matrix of size “trials” × “neurons,” where each row contained the spike counts from one trial for all neurons in the population, and the corresponding stimulus frequency label served as the target class, irrespective of the stimulus SPL level. For each unit in a pseudopopulation and for each stimulus condition, two-thirds of the trials were randomly assigned to a training set, while the remaining one-third was held out as a test set. The trained classifier was then used to predict the labels of the test set trials. For each observation, the predicted class label was determined by selecting the class associated with the smallest average binary loss, computed using a hinge loss function as part of a loss-weighted decoding strategy. Decoding performance was quantified as the percentage of correctly classified test trials, computed both as an overall average across all stimulus frequencies, for each frequency separately and individually for each frequency–amplitude combination. To establish a baseline for chance-level performance, the same decoding procedure was repeated on datasets in which the stimulus frequency labels were randomly shuffled across trials. This process was conducted independently for each pseudopopulation. To further assess the generalizability of stimulus representations across protocols, we implemented a decoder trained on one condition (PT or NBN) and tested it on the other. For this analysis, pseudopopulations were built exclusively from units that were responsive to both conditions, and all available trials from the training condition were used to build the model. The overall structure of this decoder followed the same multiclass SVM framework described above. Hyperparameter optimization was performed prior to cross-validation, and classifier performance during training was estimated via stratified cross-validation to ensure balanced representation across frequency bands. Test performance was then quantified by applying the trained model to the responses of the same pseudopopulation when presented with the trials of the other condition. Predictions and baseline performances were computed using the same loss-weighted decoding strategy as in the within-condition multiclass SVM.

#### Statistical analysis

Statistical analyses were performed using parametric or nonparametric tests depending on the distributional properties of the data. Unless otherwise stated, statistical significance was evaluated at *α* = 0.05 using two-tailed tests. Exact *p*-values, test statistics, and sample sizes are reported in the Results or in the corresponding figure legends. For analyses involving multiple comparisons, appropriate corrections were applied as specified in the relevant sections. All statistical procedures were implemented in MATLAB.

## Results

**Figure 1.**
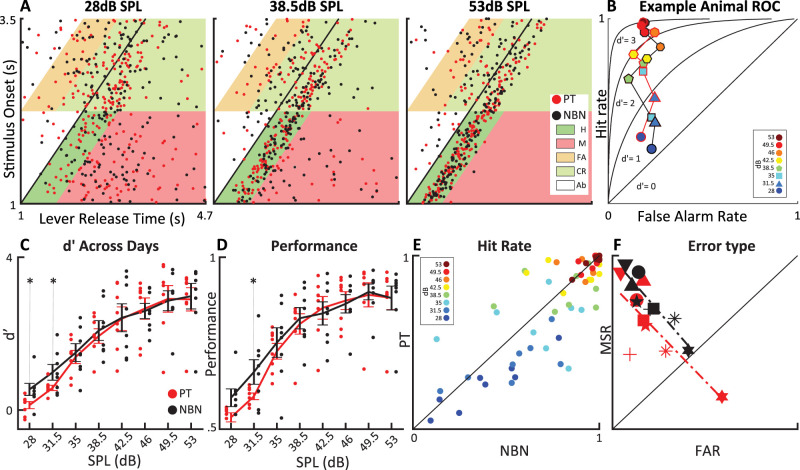
Stimulus-dependent behavioral effects in the detection paradigm. ***A***, Lever release times for a representative animal at low, medium, and high sound pressure levels (SPLs). The black oblique line marks the target onset. Colored trapezoids indicate different trial outcomes: hit (H), miss (M), false alarm (FA), correct rejection (CR), and abort (AB), as defined in the legend. For individual animal performance, see Extended Data Figures 1-1–1-3. ***B***, False alarm and hit rates are plotted across different amplitudes, with each amplitude represented by a distinct marker: Circles denote the lowest amplitude, and markers with increasing numbers of polygonal sides represent progressively higher amplitudes, for the same animal as in ***A***. The consistency of the false alarm (FA) rate across different amplitudes suggests that the behavior is associated with the waiting for the stimulus and the consistency of the animal's strategy. ***C***, Comparison of mean *d*′ values across all behavioral sessions (*n* = 10) for all animals (*n* = 8) in response to PT and NBN stimuli. The * denotes significance of a pairwise *t* test Tukey–Kramer corrected *p* < 0.05. ***D***, Comparison of mean performance values, showing similar results to the *d*′ measure. ***E***, The HR (average across 10 sessions) is higher for the NBN condition at all amplitudes, and it's significant for the lowest two (paired *t* test 28 dB *p* < 0.01, 31.5 dB *p* < 0.05). ***F***, Each animal shows a negative correlation between FAR and MSR at the lowest amplitude for both stimulus sets (*t* test PT *p* < 0.01, NBN *p* < 0.05), with no significant difference in the linear fits between them (Fisher's test, *p* > 0.05). As expected, animals with higher MSR tend to have lower FAR for both stimuli. The animal represented by the square corresponds to the example shown in ***A*** and ***B***.

To investigate the perception of PT and NBN auditory stimuli in rats, we employed a detection task to assess behavioral sensitivity to target sounds at eight different amplitudes. Rats were trained to press a lever and release it upon presentation of a target sound (NBN, 7–10 kHz; PT, 8.5 kHz; see Materials and Methods). For precise quantification of detection performance, we computed *d*′ as well as percent correct performance (Wang et al., 2023). Example behavioral data for a single animal aggregated across the last 10 d of task performance for target amplitudes of 28, 38.5, and 53 dB are shown in Figure 1*A* (for the complete individual animals’ performance, see Extended Data Figs. 1-1–1-3). As expected, the animals’ performance improved with increasing target amplitude. Figure 1*B* plots hit rate (HR) versus false alarm rate (FAR), as well as the ROC curves for four values of *d*′ for the same animal as in A. While HR and *d*′ increase systematically with target amplitude, FAR remains stable for both PT and NBN stimuli. This is expected for our analysis method, because no target sound occurred prior to the end of the false alarm response window, and therefore by definition the FAR cannot depend on target amplitude. A two-way ANOVA across all animals (*n* = 8) revealed a robust amplitude dependence of *d*′ and performance for both stimulus types, *p* < 0.05 (Fig. 1*C*,*D*). For each animal, *d*′ and performance values were first averaged across 10 behavioral sessions to obtain a subject-level estimate for each condition, and these subject-level means were then averaged across animals to yield the group-level values. Notably, animals showed a higher sensitivity to the NBN compared with the PT stimuli, reflected in significantly higher *d*′ values at the two lowest amplitudes (Fig. 1*C*, paired *t* test Tukey–Kramer corrected *p* < 0.05). At the same time, the *d*′ for PT at the lowest amplitude did not differ from zero (*t* test, *p* > 0.1). Together, this suggests that rats’ detection thresholds for PT and NBN targets were close to 31.5 and 28 dB, respectively. Consistent with this, performance as measured by percent correct at 31.5 dB was significantly better in response to NBN (Fig. 1*D*, paired *t* test Tukey–Kramer corrected *p* < 0.05). Thus, rats more accurately detected low-amplitude target sounds for NBN compared with PT stimuli, and *d*′ appears to be somewhat more sensitive than performance, highlighting the usefulness of the signal detection framework. Consistent with these findings, we observed significantly higher HRs for NBN compared with PT at the lowest two amplitudes (Fig. 1*E*, paired *t* test 28 dB *p* < 0.01; 31.5 dB *p* < 0.05). In terms of individual differences among participant rats, we found substantial variation between the two error types (Fig. 1*F*). While some rats (e.g., downward triangle) made few “false alarm” but many “miss” errors, others (e.g., hexagram) showed the opposite pattern with a preponderance of “false alarm” errors. For both PT and NBN, we observed an anticorrelation between “miss rate” (MSR) and FAR, as quantified by linear regression fits and evaluated using an *F*-statistic (*t* test PT: *R*^2^ = 0.843; *p* < 0.01. NBN: *R*^2^ = 0.616; *p* < 0.05). We consider the error type to be related to the individual rats’ response strategy, and this provides interesting supplementary information that could be useful in the context of preclinical investigations. Taken together, the rats’ auditory detection performance depended strongly on target sound amplitude for both PT and NBN stimuli. At high amplitudes, behavioral performance on both tasks reached a plateau at similar values of *d*′ ≈ 3.3 and performance ≈ 0.9. At low amplitudes, near perceptual threshold, performance, and *d*′ values were significantly higher for NBN compared with PT stimuli, suggesting that rats were more sensitive to NBN targets.

10.1523/ENEURO.0347-25.2026.f1-1 Figure 1-1 By displaying the holding time of each animal at each amplitude, it is possible to observe the distinct strategies developed and adopted by each individual. Nonetheless, all animals showed a similar overall trend: a decrease in correct responses and an increase in errors as the stimulation amplitude was lowered. Download Figure 1-1, TIF file.

**Figure 2.**
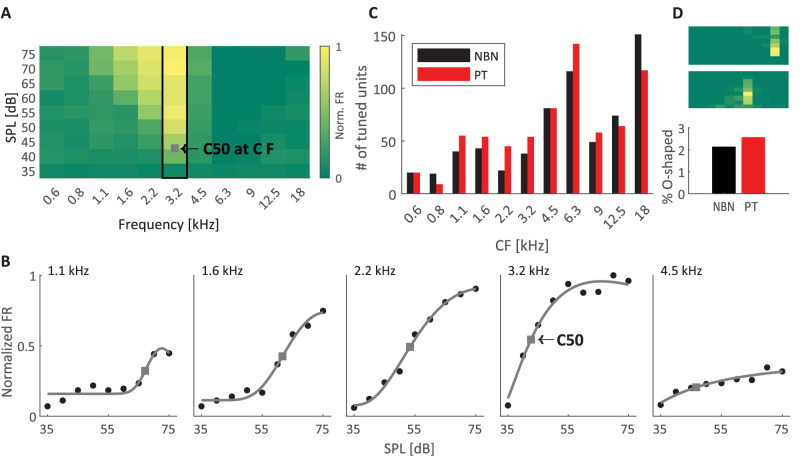
Characterization of frequency tuning to pure tones and narrowband noise in the auditory thalamus. ***A***, Example FRA from a single unit in response to NBN. The color scale represents the normalized firing rate (FR). The highlighted column indicates the unit's CF, defined as the frequency corresponding to the lowest C50 value (gray square), i.e., the SPL eliciting 50% of the maximum response. ***B***, Example of log-normal (LN) fits (gray lines) of normalized firing rates (black dots) across SPLs at selected frequencies (based on fit quality) for the same unit shown in ***A***. The C50 value is indicated for each frequency; the lowest C50 value determines the CF (3.2 KHz). ***C***, Distribution of characteristic frequencies (CFs) for neurons tuned to PT (red bars) and NBN (black bars) stimuli. The distribution is similar across both stimulus types, with most responsive units exhibiting CFs ∼6.3 and 18 kHz, and relatively few tuned below 1 kHz. ***D***, Example FRAs of units exhibiting O-shaped tuning profiles for NBN (top) and PT (bottom), below the proportion of O-shaped units for the two stimulus types. O-shaped units were defined by response profiles where firing rates peaked at intermediate SPLs and were reduced at both lower and higher sound intensities, quantified by the width of the LN fit (see Materials and Methods).

10.1523/ENEURO.0347-25.2026.f1-2 Figure 1-2 By displaying the holding time of each animal at each amplitude, it is possible to observe the distinct strategies developed and adopted by each individual. Nonetheless, all animals showed a similar overall trend: a decrease in correct responses and an increase in errors as the stimulation amplitude was lowered. Download Figure 1-2, TIF file.

**Figure 3.**
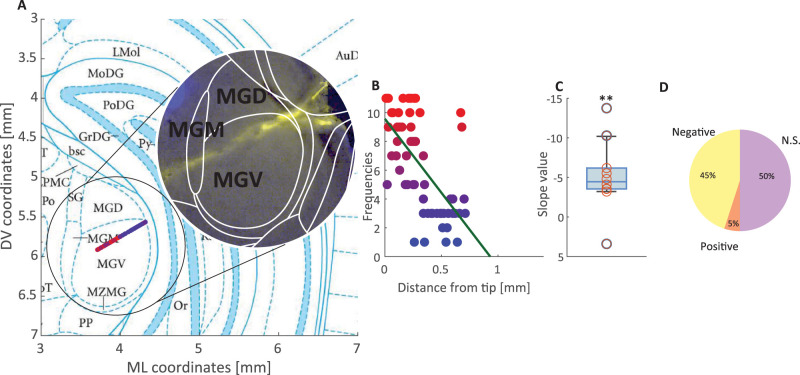
Tonotopy organization of the rat auditory thalamus (MGBv). ***A***, Schematic coronal representation of the rat MGBv showing the relative unit positions color-coded by their CFs; the scale goes from blue, low frequency, to red, high frequency. The inset on the right shows an example histological reconstruction of one penetration with the probe track shown in yellow (DiI). For reconstructions of each individual penetration, see Extended Data Figure 3-1. ***B***, A scatterplot showing CF against distance from the tip of the recorded units, with a fitted linear regression line. ***C***, ***D***, Boxplot and pie chart showing the distribution of the units with a linear regression slope significantly different from 0 (ANOVA *p* < 0.05) among all recording sessions. The average slope is significantly lower than zero (one-sample *t* test *p* < 0.01, ***C***); a result illustrated also by the pie chart proportion (***D***).

10.1523/ENEURO.0347-25.2026.f1-3 Figure 1-3 **| Single animal behavioral performances A6-A8.** Lever release times for all individual animals (A6-A8) at all amplitudes (28; 31.5; 35; 38.5; 42.5; 46; 49.5; 53 dB SPL). The black oblique line marks the target onset. Colored trapezoids indicate different trial outcomes: dark-green hit (H), red miss (M), yellow false alarm (FA), light green correct rejection (CR), and white abort (AB). Download Figure 1-3, TIF file.

To examine the neural underpinnings of these behavioral results, we performed extracellular recordings using high-density arrays in the medial geniculate body (MGB) of the thalamus in anesthetized rats (Steinmetz et al., 2021). We presented brief PT and NBN stimuli with matched frequencies in a range from 500 to 22 kHz at eight amplitudes and recorded a total of 895 auditory-responsive units (see Materials and Methods), with 699 and 653 units responding to individual PT and NBN protocols, respectively, and 449/895 (49%) responding to both protocols. We constructed the FRA (frequency response area) for each unit by calculating the average neural response across trials for both PT and NBN at each frequency–amplitude combination (Fig. 2*A*, example unit). For each frequency, we then fit a log-normal distribution (LN) to the response across amplitudes (Fig. 2*B*) and identified the C50 value that represents the sound amplitude required to elicit 50% of the unit's maximum response at that frequency. This C50 value allowed us to quantify sensitivity to sounds at each frequency for each unit. To determine the overall sensitivity of the unit, we defined the sensitivity level as the lowest C50 value observed across all tested frequencies. The frequency corresponding to this minimum C50 was designated as the unit's characteristic frequency (CF), providing a key metric for understanding the unit's optimal tuning in response to auditory stimuli. The distribution of CFs spanned the entire range of frequencies used and was equivalent between NBN and PT stimulus sets (two-sample Kolmogorov–Smirnov test, *p* > 0.1; Fig. 2*C*). Note that relatively few units had CFs at low frequencies, below 1 kHz, consistent with previous studies of MGB tonotopy (Shiramatsu et al., 2016). The LN fit of the frequency FRA enabled us to identify units exhibiting an O-shaped response profile characterized by inhibition at high amplitudes at the CF (Fig. 2*D*, examples, top panel). Our analysis revealed that a similar fraction of units (18 units, 3.5%; 14 units, 2.8%) exhibited O-shaped tuning in the PT and NBN protocols, respectively (chi-square test, *p* > 0.1; Fig. 2*D*, bottom panel). These fractions are slightly lower than previously reported values in the mouse cortex of ∼8% (Rothschild et al., 2010), suggesting that O-shaped tuning might be further elaborated in cortical circuits but is already present in thalamic auditory structures (but see Bartlett and Wang, 2011). We note that LN fits can reliably capture O-shaped tuning, which might be useful in the context of characterizing dysfunctions in auditory disorders as they involve local inhibitory interactions (Bartlett and Wang, 2011). We used the CF of each recorded unit to reconstruct MGB tonotopic organization (Fig. 3*A*), revealing a lateral-to-medial progression in CF values within the MGB (Fig. 3*B*), consistent with the well-documented tonotopic organization reported in previous studies (Bordi and LeDoux, 1994; Shiramatsu et al., 2016). Evidence for tonotopic MGB organization was observed in 9/22 of electrode penetrations with a significant negative correlation between recording site depth and CF, consistent with the MGBv auditory thalamic subfield (Fig. 3*C*). Only 1/22 penetrations exhibited a significant positive correlation that likely contains a small portion of the auditory subfield MGBm (for reconstructions of these 10 penetrations, see Extended Data Fig. 3-1).

10.1523/ENEURO.0347-25.2026.f3-1 Figure 3-1 **| Tonotopic organization of the rat auditory thalamus (MGB). A.** Schematic coronal representation of the rat MGB showing the relative unit positions, color coded by their CFs, for all the penetration with a significant recorded response; the scale goes from blue, low frequency, to red, high frequency. **B.** A scatterplot showing CF against distance from the tip of the probe for the recorded units, with a fitted linear regression line. Download Figure 3-1, TIF file.

**Figure 4.**
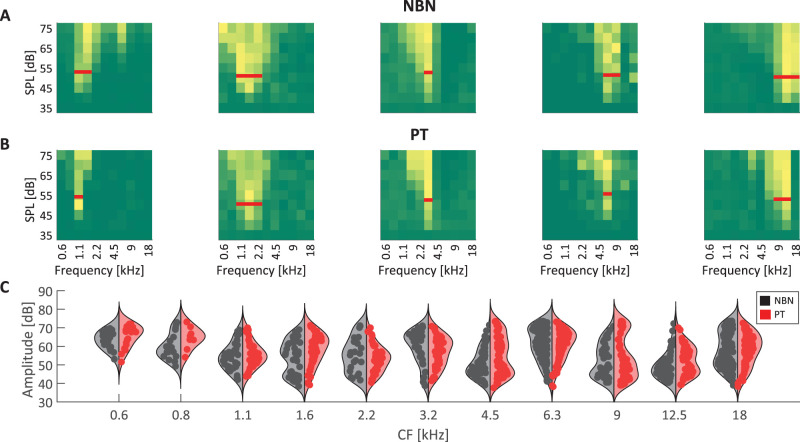
Auditory tuning properties in MGB. ***A***, Example FRAs from individual MGB units in response to NBN stimuli with different CF. Color scale indicates normalized firing rate as a function of stimulus frequency (*x*-axis) and sound pressure level (SPL, *y*-axis). The red horizontal line marks the tuning width, computed at 10 dB above each unit's C50 level. ***B***, Example FRAs from the same units in response to PT stimuli. No significant difference in width was found between the two stimulus types (*p* > 0.1, Mann–Whitney *U* test). ***C***, Distribution of C50 values for all recorded units as a function of their CF, separately for NBN (black) and PT (red) conditions. Each violin plot represents the distribution of C50 values at a given frequency band, with individual data points overlaid. A two-way ANOVA revealed a significant main effect of stimulus type on C50 values (*p* < 0.01, 2-way ANOVA), demonstrating how NBN produces higher sensitivities.

**Figure 5.**
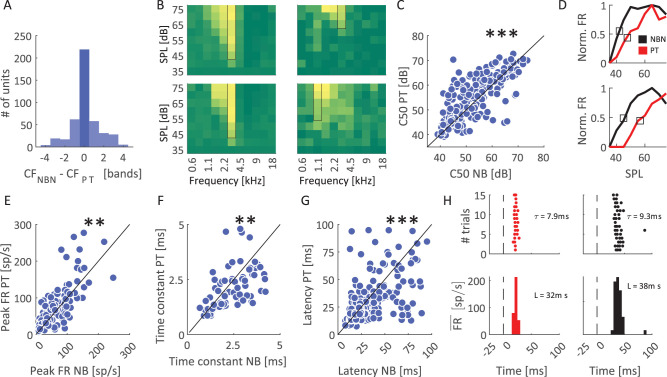
Comparison of response properties to pure tones and narrowband stimuli in auditory thalamic neurons. ***A***, Distribution of the difference in characteristic frequency (CF) between NBN and PT for units responsive to both protocols (*n* = 449). Most units exhibited minimal or no CF shift. ***B***, Example FRAs for two units illustrating cases without (left) and with (right) CF shifts between NBN (top) and PT (bottom) stimuli. ***C***, Scatterplot comparing C50 values between PT and NBN for individual units. The diagonal line corresponds to no shift in C50 between the two stimulus classes. C50 values were strongly correlated but significantly lower for NBN (***p < 0.001, paired Wilcoxon signed-rank test). ***D***, Example response functions at CF for two representative units. Normalized firing rates (Norm. FR) are plotted against SPL, with NBN responses showing higher thresholds and steeper slopes. ***E***, Scatterplot displaying the difference in peak FR between PT and NBN. Our analysis highlighted how PT produced a higher peak FR than NBN (**p < 0.01, paired Wilcoxon signed-rank test). ***F***, Scatterplot comparing time constants of neuronal responses, derived from exponential fits to ISI distributions, between PT and NBN. Responses to NBN exhibited significantly longer time constants (**p < 0.01, paired Wilcoxon signed-rank test). ***G***, Comparison of response latencies, quantified as the time to peak in the PSTH at the best stimulus (stimulus eliciting the highest average firing rate at CF). NBN stimuli produced significantly longer latencies than PT (***p < 0.001, paired Wilcoxon signed-rank test). ***H***, Example PSTHs from two representative neurons. Top, Raster plots showing spike times across trials for NBN (black) and PT (red) stimuli. Bottom, Corresponding PSTHs illustrating delayed and prolonged responses to NBN relative to PT ( $\bar{\mathrm{FR}}$: average firing rate across trials). Vertical black dashed line indicates stimulus onset.

We next characterized the tuning properties of individual MGB units in response to both PT and NBN stimuli. This analysis was performed on all responsive units, regardless of whether they responded to only PT, only NBN, or both stimulus types. Example FRA from single units illustrates the range of frequency selectivity observed in response to NBN and PT (Fig. 4*A*,*B*). To quantify the receptive field width, we measured the bandwidth 10 dB above the C50 level for each unit, observing no significant difference in receptive field width between the two stimulus types (*p* > 0.1, Mann–Whitney *U* test). Examining the receptive field width at each amplitude, irrespective of relation to C50 level following Kajikawa et al. (2011), we found overall significantly narrower values for PT compared with NBN targets at 60 dB (Wilcoxon rank-sum test, *p* < 0.05), providing some evidence for more precise information conveyed by PT stimulation. We then grouped units based on their CF and compared the distribution of C50 values, derived from the FRA (Fig. 4*C*). We identified significant main effects of frequency and stimulus type (two-way ANOVA, *p* < 0.01), such that MGB units exhibited reduced sensitivity at low frequencies, particularly those under 1 kHz, and were on average more sensitive to NBN than PT stimuli, with median C50 values of 55.5 and 57.7 dB respectively. This mirrors our behavioral findings, where detection performance of rats was also greater for NBN than PT stimuli near the behavioral auditory threshold, suggesting that sensory signals in the auditory thalamus convey information about low-amplitude sounds that rats can utilize to optimize behavioral performance. To further quantify and compare neuronal responses to PT and NBN, we assessed multiple response properties only in the subset of units responsive to both stimulus protocols (*n* = 449). First, we wanted to assess whether there was any systematic shift in CF between the two protocols. The distribution of CF differences between PT and NBN was symmetric and centered at zero, with 49% of units showing no shift in CF between the two stimulus types (Fig. 5*A*), and there was no significant difference on average (paired *t* test, *p* = 0.07), confirming there was no systematic shift in characteristic frequency preference between the two stimulus protocols. Representative FRAs are shown in Figure 5*B*, illustrating an example unit with consistent CFs between PT and NBN, as well as another unit with a CF shift. It thus seems that both protocols are useful for determining MGB frequency tuning and yield overall coherent CF values. Next, we assessed whether units were differentially sensitive to PT and NBN, using the subset of units that maintained consistent CF across both protocols (*n* = 219). While C50 values were overall strongly correlated between stimulus types (*ρ* = 0.74, *p* < 0.001), C50 values were significantly lower for NBN compared with PT stimulus across the MGB population (paired Wilcoxon signed-rank test, *p* < 0.001; Fig. 5*C*), suggesting that MGB units with consistent CF values and responsive to both protocols were generally more sensitive to NBN compared with PT stimuli, consistent with results in Figure 4*C* for the entire population of units. Representative tuning curves for two example units illustrate this effect, with NBN responses requiring lower SPLs to elicit similar firing rates (Fig. 5*D*). Next, we wanted to assess if there were overall changes in unit responsivity between the two protocols. Examining the entire response, we did not observe a significant difference in median firing rate in response to the amplitude eliciting the highest activity at the CF between PT and NBN (median firing rates: PT 19.5, NBN 19.6; paired Wilcoxon signed-rank test, *p* > 0.1). However, examining the time course of the neural response with 5 ms temporal resolution, PT triggered higher peak activity than NBN stimuli (median peak FR: NBN = 34.1 sp/s, PT = 39.3 sp/s; paired Wilcoxon signed-rank test, *p* < 0.001; Fig. 5*E*). In light of the observed difference between average and peak firing rates, we next assessed the persistence of neural responses by estimating time constants derived from exponential fits to interspike interval (ISI) distributions following Matteucci and colleagues (Murray et al., 2014; Matteucci and Zoccolan, 2020). For units with reliable fits (*R*^2^ > 0.7), the time constants were significantly lower for PT stimuli, reflecting faster temporal dynamics and response adaptation compared with NBN stimuli (paired Wilcoxon signed-rank test, *p* < 0.01; Fig. 5*F*). At the same time, response latencies, computed as the distance in time from stimulus onset to the peak of the peristimulus time histogram (PSTH), were shorter for PT compared with NBN stimuli (median latency PT 23 ms, NBN 26 ms; paired Wilcoxon test, *p* < 0.001; Fig. 5*G*). Neural spiking activity of an example MGB unit illustrates shorter latency and faster temporal dynamics for PT stimuli (Fig. 5*H*). Taken together, while activations to PT and NBN exhibited substantial similarities, PT-evoked responses exhibited shorter response latencies, faster temporal dynamics as well as more rapid response adaptation, while units were more sensitive to NBN stimuli. Importantly, these timing measures were compared at the amplitude that elicited the highest activity at the CF for each respective stimulus (PT or NBN). Although the amplitude eliciting the highest activity may differ between PT and NBN, we used this approach to maximally drive each unit for the respective stimulus, thus providing the least biased comparison. Notably, for approximately 70% of the units included in this analysis, the preferred amplitudes for PT and NBN differed by no more than ±5 dB.

**Figure 6.**
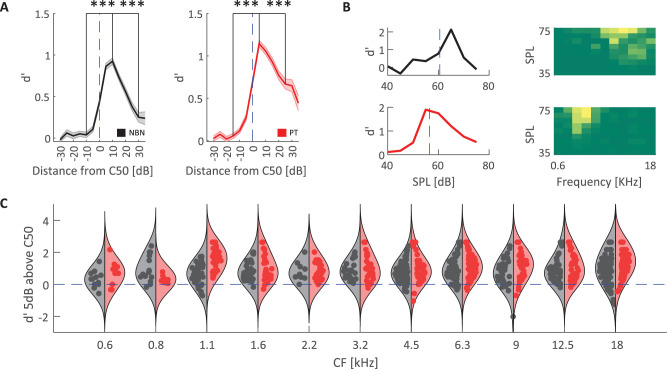
Discriminability (*d*′) of narrowband noise (NBN) and pure tone (PT). ***A***, Mean *d*′ values as a function of distance from each unit's C50. NBN (left, black) and PT (right, red) both show a sharp drop in *d*′ for stimuli ±20 dB from *d*′ peak situated 5–10 dB above C50 (***p < 0.001, paired *t* tests). Shaded areas represent SEM. ***B***, Example *d*′ values across amplitudes for two individual units responsive to NBN (top) and PT (bottom); blue dashed lines indicate the unit's C50. Corresponding FRAs are shown on the right. Examples of single-animal *d*′ distributions are shown in Extended Data Figure 6-1. ***C***, Violin plots showing the distribution of *d*′ values computed 5 dB above C50 across sound levels for units responsive to both NBN (black) and PT (red), plotted as a function of CF. Each violin represents a frequency band, with individual unit data overlaid. A significant main effect of both frequency and stimulus type was found (*p* < 0.001, 2-way ANOVA), with lower *d*′ values for frequencies below 1 kHz and higher *d*′ for PT compared with NBN across frequencies.

So far, we have focused on auditory target detection performance and related aspects of thalamic neural circuit activation. Another important aspect of auditory function is the ability to discriminate among sounds of different frequencies. Along these lines, we performed an analysis examining the capacity of single MGB neurons responsive to both NBN and PT to discriminate between sounds at their CF from adjacent frequencies, using a signal detection framework similar to the one we used for quantifying behavioral performance (see Materials and Methods). For each unit, we computed *d*′ at the C50 amplitude as well as amplitude values below and above, in order to assess the neuron's ability to communicate frequency-specific information around its response threshold. For both NBN and PT (Fig. 6*A*), neural sensitivity (*d*′) exhibited a peak at sound amplitudes 10 and 5 dB above C50, respectively, suggesting maximum frequency sensitivity occurred for sound amplitudes slightly above the response threshold. For sound amplitudes lower than C50, *d*′ values were close to zero, since neurons were largely unresponsive. Interestingly, increasing sound amplitudes beyond 10 dB above C50 did not enhance *d*′ values, but actually led to reductions in neural sensitivity (paired *t* test comparing *d*′ values at the peak to values at amplitude peak + 20 dB, *p* < 0.001). We suggest that this reduced *d*′ at higher amplitudes is due to a broadening of the auditory tuning function that occurs away from the tip of the tuning curve, such that adjacent frequencies also trigger notable neural responses that reduce single neuron frequency discriminability. This effect is illustrated in Figure 6*B*, showing single-unit examples *d*′ sensitivity across SPL and associated frequency response areas, consistent with the population average in Figure 6*A* (examples of single-animal *d*′ distributions are shown in Extended Data Fig. 6-1). Plotting the distribution of *d*′ at C50 + 5 dB across CF values (Fig. 6*C*), we find significant main effects of frequency and stimulus type (two-way ANOVA, *p* < 0.05), with higher *d*′ values for PT than NBN targets and reduced *d*′ values below 1 kHz for both stimulus types.

10.1523/ENEURO.0347-25.2026.f6-1 Figure 6-1 **| Discriminability (d’) of narrowband noise (NBN) and pure tone (PT) single animals. A**-**D.** Mean d’ values as a function of distance from each unit’s C50 for 2 example animal (animal 3 – A, animal 4 - D). NBN (left, black) and PT (right, red) both show a sharp drop in d’ for stimuli ± 20 dB from d’ peak situate 5-10 dB above C50 (p < 0.001, paired t-tests). Shaded areas represent SEM. **B**-**E.** Examples coming from 2 different subjects (animal 3 – B, animal 4 - E) of d’ values across amplitudes for two individual units responsive to NBN (top) and PT (bottom), blue dashed lines indicate unit’s C50. Corresponding FRA are shown on the right. **C**-**F.** Violin plots showing the distribution of d’ values computed 5 dB above C50 across sound levels for units responsive to both NBN (black) and PT (red), plotted as a function of characteristic frequency (CF) for 2 example animal (animal 3 – C, animal 4 – F). Each violin represents a frequency band, with individual unit data overlaid. A significant main effect of both frequency and stimulus type was found for animal 3 while we only found a main effect of frequency for animal 4 (two-way ANOVA, C: p < 0.001 – frequency and p < 0.05 – stimulus type; F: p < 0.05 – frequency and p > 0.1 – stimulus type). **G**. Summary figure showing for each subject in which frequency bands the average peak d’ exceeded 1 across the recorded units for NBN (left) and PT (right). Download Figure 6-1, TIF file.

**Figure 7.**
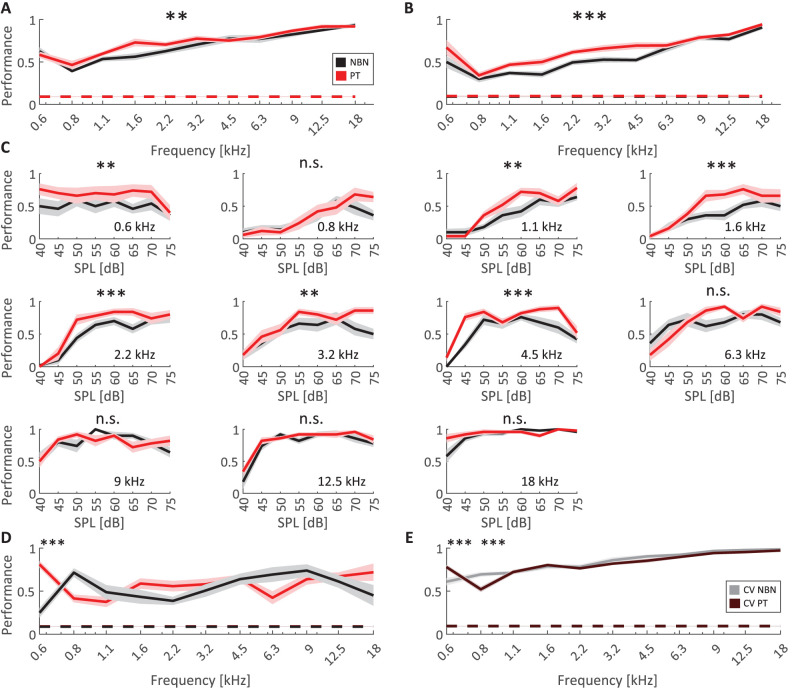
Decoding performance for frequency discrimination. ***A***, ***B***, Multiclass SVM decoding performance for predicting NBN and PT stimulus frequency based on spike count responses from populations of 50 neurons (average across 10 populations for each condition; see Extended Data Fig. 7-1 for a comparison of the effects of population size). Performance has been evaluated for two temporal windows: the longer response window (0–50 ms, ***A***) used in *d*′ analysis and an early response window (0–25 ms, ***B***) to investigate the impact of the differences in response timing. ***A***, During the 50 ms response window, decoding accuracy was significantly different between PT and NBN, with PT achieving higher performances especially in the 0.8–3.2 kHz interval (2-way ANOVA). We also observed a significant increase in decoding accuracy with frequency (2-way ANOVA). ***B***, In the early response window (0–25 ms), decoding performances were even higher for PT (red) compared with NBN (black) stimuli across frequencies (*p* < 0.001, 2-way ANOVA). Shaded areas represent ± SEM across the 10 populations. Horizontal dashed line indicates chance level (∼9.1%, based on shuffled labels). ***C***, Decoding performance as a function of sound pressure level (SPL) for a given center frequency, comparing NBN and pure tone PT stimuli using the early response window as in ***B***. A two-way ANOVA revealing significant effects of stimulus type, with PT stimuli consistently yielding higher decoding accuracy than NBN. No significant effect of the stimulus type was observed at other frequencies. Examples of decoding analyses from units of individual animals are shown in Extended Data Figures 7-2 and 7-3. ***D***, Cross-condition decoding performance for classifiers trained and tested on different stimulus types. Red: classifier trained on NBN and tested on PT. Black: classifier trained on PT and tested on NBN. A two-way ANOVA on frequency and stimulus type revealed a significant interaction. Further multiple-comparisons test revealed significant differences in decoding performance at the lowest amplitude (pairwise *t* test Tukey–Kramer corrected). ***E***, Within-condition cross-validation performance on the training data. Gray: classifier trained and cross-validated on NBN; brown: classifier trained and cross-validated on PT. High performance in both cases indicates successful training, suggesting that the drop in ***D*** reflects reduced generalization across stimulus types rather than poor model fit. Using the same analysis as in ***D***, revealed differences in decoding performance at the two lowest amplitudes (pairwise *t* test Tukey–Kramer corrected). In all panels: *p < 0.05, **p < 0.01, ***p < 0.001.

Beyond the single-unit related analyses above, the large number of units that can be collected from individual animals also permits population analyses, revealing how much information about auditory stimuli is carried by the population of recorded units. To examine neuronal population activity, we trained multiclass SVM classifiers on 11-class frequency discrimination based on spike count responses at all amplitudes from randomly assembled populations of 50 neurons using 10 independent populations per condition separately for PT and NBN stimuli (see Materials and Methods). Decoding performance was quantified as the percentage of correctly classified test trials (1/3 of total trials), averaged across the 10 populations. We repeated the analysis using two temporal windows: 0–50 ms following target onset for consistency with the above *d*′ analyses and an early window (0–25 ms, corresponding to the average peak-latency) to explore the differences in response dynamics that emerged during the previous latency and slowness analyses. Observed classification performances were above chance levels across all frequencies and temporal windows (chance level 9.1% for 11 classes using shuffled labels; Fig. 7*A*,*B*) and exhibited an increasing trend across frequencies. For both windows, there were significant main effects of frequency and stimulus type (two-way ANOVAs, *p* < 0.01), with classification using PT responses yielding higher performance than NBN responses and a more pronounced PT advantage occurring in the early response window. Together, these population analyses show that classification performance increases with frequency in the range tested and can reach values of ∼0.9 performance at 18 kHz. PT responses permit significantly higher classification accuracy, particularly in an intermediate frequency range between ∼1 and 6 kHz. To assess the effect of target amplitude on classification performance, we show the classification accuracy achieved at each separate amplitude of all 11 frequencies (Fig. 7*C*). These analyses illustrate that classification performance (1) was low and often near chance at 40 dB, (2) tended to increase systematically with amplitude, and (3) was often higher for PT than NBN stimuli at intermediate amplitudes (examples in Extended Data Figs. 7-2 and 7-3 confirm these observations at the single-animal level). Lastly, we examined cross-condition classification performance, where the classifier was trained on PT responses and tested on NBN responses and vice versa (Fig. 7*D*). We observed that in this condition, the classifier nevertheless achieved good performance of 0.5 or greater across frequencies that was well above chance, with a significant difference in classification performance between PT and NBN (two-way ANOVA interaction term *p* < 0.01). This interaction appeared complex however, for example, with an advantage for PT at 600 Hz and for NBN at 800 Hz. Importantly, the reduced accuracy relative to within-condition decoding does not reflect poor model fitting: As shown in Figure 7*E*, within-condition performance was directly assessed here using stratified fivefold cross-validation applied only to the training data, providing an unbiased estimate of how well each classifier performs when tested on held-out folds from the same stimulus type. Within-condition accuracy remained high for both PT and NBN, confirming that the decoders were well-trained and that the decrease observed in cross-protocol testing reflects only partial generalization across stimulus types. Together, these findings indicate that while PT- and NBN-evoked population responses are not fully interchangeable, they are sufficiently similar to support above-chance generalization across stimulus classes.

10.1523/ENEURO.0347-25.2026.f7-1 Figure 7-1 | Decoding performance as a function of population size across frequencies. Decoding accuracy is shown for increasing numbers of units at different center frequencies, comparing NBN (black) and PT (red) stimuli using the 0–50 ms response window. Error bars denote ± SEM across 10 random populations. The vertical dashed blue line marks a population size of 50 units, which was used for all main decoding analyses. At most frequencies, performance improved steeply with population size up to ∼50 units, after which gains plateaued and additional units contributed little to further improvement. Download Figure 7-1, TIF file.

10.1523/ENEURO.0347-25.2026.f7-2 Figure 7-2 **| Decoding performance for frequency discrimination animal 3. A-B.** Multiclass SVM decoding performance for predicting NBN and PT stimulus frequency based on spike count responses from populations of 50 neurons (average across 10 populations for each condition). Performance has been evaluated for two temporal windows: the longer response window (0-50 ms, A) used in d’ analysis, and an early response window (0-25 ms, B) to investigate the inpact of the differences in response timing. **A.** During the 50 ms response window, decoding accuracy was significantly different between PT and NBN, with PT achieving higher performances (p < 0.05, two-way ANOVA). We also observed a significant increase of decoding accuracy with frequency (p < 0.001, two-way ANOVA). **B**. In the early response window (0-25 ms), decoding performances were even higher for PT (red) compared to NBN (black) stimuli across frequencies (p < 0.001, two-way ANOVA). Shaded areas represent ± SEM across the 10 populations. Horizontal dashed line indicates chance level (∼9.1%, based on shuffled labels). **C.** Decoding performance as a function of sound pressure level (SPL) for a given center frequency, comparing NBN and pure tone PT stimuli using the early response window as in B. A two-way ANOVA revealed a significant effect of stimulus type at 560 Hz (p < 0.05), 800 Hz (p < 0.001), 1120 Hz (p < 0.01), 1600 Hz (p < 0.01), with PT stimuli consistently yielding higher decoding accuracy than NBN. No significant effect of the stimulus type was observed at other frequencies. Download Figure 7-2, TIF file.

10.1523/ENEURO.0347-25.2026.f7-3 Figure 7-3 **| Decoding performance for frequency discrimination animal 4. A-B.** Multiclass SVM decoding performance for predicting NBN and PT stimulus frequency based on spike count responses from populations of 50 neurons (average across 10 populations for each condition). Performance has been evaluated for two temporal windows: the longer response window (0-50 ms, A) used in d’ analysis, and an early response window (0-25 ms, B) to investigate the inpact of the differences in response timing. **A.** During the 50 ms response window, decoding accuracy was not significantly different between PT and NBN (p = 0.053, two-way ANOVA). We observed a significant increase of decoding accuracy with frequency (p < 0.001, two-way ANOVA). **B**. In the early response window (0-25 ms), decoding performances were higher for PT (red) compared to NBN (black) stimuli across frequencies (p < 0.001, two-way ANOVA). Shaded areas represent ± SEM across the 10 populations. Horizontal dashed line indicates chance level (∼9.1%, based on shuffled labels). **C.** Decoding performance as a function of sound pressure level (SPL) for a given center frequency, comparing NBN and pure tone PT stimuli using the early response window as in B. A two-way ANOVA revealed a significant effect of stimulus type at 1120 Hz (p < 0.01), 1600 Hz (p < 0.01), 2240 Hz (p < 0.001), 3150 Hz (p < 0.01), with PT stimuli consistently yielding higher decoding accuracy than NBN. No significant effect of the stimulus type was observed at other frequencies. Download Figure 7-3, TIF file.

## Discussion

Our behavioral results reveal that rats were systematically better able to detect 7–10 kHz NBN target sounds compared with PT targets at the corresponding center frequency of 8.5 kHz. Although our behavioral experiments focused on a single frequency band (8.5 kHz), our neural data indicate that the higher sensitivity for NBN stimuli did not appear to be specific to this band but was generally observed across the range of frequencies studied. A two-way ANOVA on C50 values at CF revealed significant main effects of both stimulus type and frequency, but no significant interaction (Fig. 4), demonstrating a consistent advantage for NBN stimuli across frequencies without systematic reversals of stimulus preference. Thus, although absolute behavioral sensitivity is expected to vary with frequency, the relative advantage of NBN over PT stimulation is likely to extend across the tonotopic axis. The differences in sensitivities we observed are compatible with findings in humans. For example, a study in young adults with normal hearing found that NBN thresholds were systematically lower than PT thresholds across a broad range of frequencies, 0.5–16 kHz (John et al., 2017). Differences in threshold ranged from 3 to 5 dB for lower frequencies, up to ∼8 kHz, and increased to up to 8 dB at the high end of the frequency range tested. These values correspond well to our results in the rat, which yielded a threshold difference of 3.5 dB in the 7–10 kHz range. A similar threshold difference was recently reported in human subjects (3.2 dB) using an NBN with a 1/3-octave bandwidth (Buss et al., 2024), which is similar in spectral extent to the 1/2-octave bandwidth employed in our study. Our findings reveal a homology between human and rat auditory detection performance, which is an important prerequisite for translating preclinical animal work toward applications in humans. Accentuated lower thresholds for NBN compared with PT targets can be observed in humans with hearing impairments, and these threshold differences tend to increase with steeply sloping hearing loss where they can reach values of 20 dB or more (Norrix and Anderson, 2015). It is thought that this effect is due to subjects using sound contained in the NBN stimuli at spared frequencies, thereby compensating for a specific loss of hearing in a narrow range that is more precisely assessed with PT stimuli. Interestingly, comparisons between PT and NBN stimuli play a role in behavioral diagnosis of tinnitus, where it is thought that the spontaneously generated sound sensations consist of a prominent center frequency with some spectral spread and can therefore be better approximated by NBN than PT sounds (Korth et al., 2021). The spectral spread of tinnitus identified in human studies often ranges from 1/3 to 1 octave (Henry et al., 2013; Korth et al., 2021), suggesting that the bandwidth we employed is well within the range of commonly observed tinnitus percepts. It is possible that critical band effects (King et al., 2015) might also play a role in our findings. A critical band is a frequency range within which a tone will affect the perception of a previous tone. According to behavioral experiments conducted by Gourevitch (1965) in rats, the critical band at a center frequency of 8.5 kHz, which corresponds to the target NBN sound in our experiments, was determined to be ∼3.1 kHz, based on the observed critical ratio of 35 dB (Fig. 6, compare Gourevitch) and using the Fletcher formula CB = 10^(CR/10)^ that related critical band to critical ratio. This value is similar to the half-octave bandwidth that we used in our study, i.e., 3.0 kHz, suggesting that the critical band effects might not play a large role in our observations. However, critical band estimates can vary substantially depending on how they were estimated (Fay, 1988), such that we cannot rule out that critical bands might have contributed to our findings. Indeed, if the half-octave bandwidth substantially exceeded the critical band, adjacent sections of the cochlea might be recruited by these NBN stimuli compared with pure tones. This would be expected to produce higher perceived loudness of NBN compared with PT stimuli, as we have observed in our behavioral experiments at SPL values close to perceptual threshold.

The lower thresholds for NBN targets observed behaviorally are closely paralleled by our neural recordings in MGB, which revealed lower C50 values, i.e., higher sensitivity, for NBN compared with PT targets (Fig. 4). The magnitude of the sensitivity difference between PT and NBN reached similar values of ∼3.5 dB for behavior and 2.2 dB for the MGB neural population, which is in close correspondence and suggests that MGB recordings in anesthetized rats appear to capture behaviorally relevant activations. The use of anesthesia enabled multiple, stable, large-scale recordings within a single session (Tennigkeit et al., 1997; Ries and Puil, 1999). Anesthesia is known to influence several aspects of neural activity, including excitability, tonic versus burst firing, and correlation structure (Tennigkeit et al., 1997; Noda and Takahashi, 2015), yet important properties such as frequency tuning are only weakly affected and remain comparable to those observed in the awake state (Edeline et al., 1999; Cai et al., 2016; Gosselin et al., 2024). Available evidence suggests that anesthesia is expected to attenuate rather than exaggerate differences in neural firing across conditions (Cheung et al., 2001). Thus, the sensitivity differences we report between PT and NBN are unlikely to result from anesthesia-related enhancement and instead appear to reflect stimulus-dependent differences in MGB responses. While few previous studies have directly compared PT and NBN responses in the auditory pathway, it has been reported that in the marmoset auditory cortex NBN stimuli also evoke greater activation for low amplitude stimuli (Kajikawa et al., 2011) and similarly NBN stimuli overall elicit greater activity in macaque auditory cortical areas than matched PT stimuli (Rauschecker and Tian, 2004). Overall, in the rat NBN is more easily detected at low amplitude consistent with findings in humans, and the neural recordings in MGB highlight a similar advantage of NBN in terms of the C50 parameter.

Our data show that while MGB neural sensitivity at low amplitude is greater for NBN, PT triggers MGB activations characterized by a higher peak response rate and lower latency. For targeted analyses related to the timing of responses, the use of PT stimuli is preferable as it allows for a more accurate estimation of response timing. In addition, we show that MGB PT responses also decay with shorter time constants than NBN-triggered responses, and this effect cannot be attributed to differences in mean firing rate. The significantly slower decay time constants observed for NBN-evoked responses compared with PT-evoked responses (Fig. 5*F–H*) suggest that stimuli with broader spectral content engage circuits that sustain activity for longer periods, potentially reflecting additional integrative or recurrent processing within the auditory thalamocortical pathway. Consistent with this idea, cortical areas are thought to form a hierarchy with intrinsic timescales increasing from primary sensory to higher regions, such that areas involved in tasks requiring longer integration times exhibit slower dynamics (Murray et al., 2014). While the longer decay time constants for narrowband noise are consistent with recurrent processing, an alternative explanation for our results could also be that the shorter time constants for pure tones reflect stronger inhibitory processes immediately following the onset response, which would effectively shorten the measured response duration.

Our findings demonstrate that in the rat auditory thalamus, peak response latencies to pure tones are significantly shorter than those to NBN, indicating more rapid processing of frequency-specific stimuli at this subcortical level. This stands in contrast to findings in auditory cortical areas, where studies in macaques have observed either no difference in latency or faster responses for NBN stimuli (Lakatos et al., 2005; Kajikawa et al., 2011), with latency advantages for NBN stimuli tending to grow from primary to high-level cortical areas. The reversal in preference can be attributed to two ascending auditory pathways (Rauschecker et al., 1997; Camalier et al., 2012), such that the MGBv, which we targeted in our study, projects largely to A1 (the main recipient of PT information), whereas the NBN-dominant responses in higher cortical areas are thought to result from projections from the MGBd thalamic nucleus, which contains units with short latency responses and broad frequency tuning (Bartlett, 2013). While the possibility exists that this preference reversal is due to fundamental species differences in ascending auditory pathway organization between rodents and primates, we consider this unlikely given the established cross-species conservation of organization in the auditory thalamus (Winer, 2011; Lee, 2015). Nevertheless, further experiments in both species are required to definitively address this issue. One potential alternative reason for the longer latencies and extended response dynamics observed for NBN stimuli could be the fluctuating temporal envelope inherent to noise bands, particularly for narrower bandwidths. However, given our stimulus design and the distribution of recorded units, we consider it unlikely that stochastic NBN envelope fluctuations drive the effects we report. We used 1/2-octave NBN bands, so for center frequencies above 1 kHz the absolute bandwidth exceeds 350 Hz, placing most of the modulation energy above the temporal range that auditory neurons can reliably follow [<200 Hz (Wang et al., 2008; Bharadwaj et al., 2014)]. Furthermore, the majority of our recorded units have characteristic frequencies above 1 kHz, and latency/time constant measures were computed at each unit's CF, meaning that most neurons encounter limited low-frequency envelope modulation.

We have found that individual MGB neurons communicate a maximum of frequency-specific information ∼5–10 dB above the C50 amplitude at their CF. It is thought that units with the lowest C50 values determine the hearing threshold at each sound frequency, and the consistent results of behavioral and neuronal analyses in the present study support this notion. But what is the function of units with C50 values that are considerably elevated from the hearing threshold and which are considerably more numerous than those active near threshold? Clearly, these neurons cannot contribute to frequency discrimination close to threshold, as they are not active for these auditory stimuli. However, we suggest that they support auditory processing in the intermediate and high decibel range, where units with low C50 are no longer sensitive due to the broadening of their tuning function. As units are progressively lost during hearing loss, the perishing of relatively few neurons with near-threshold sensitivity is expected to produce notable elevations in behavioral thresholds. Conversely, a larger number of the initially more numerous units with lower sensitivity will continue to function for processing of larger amplitude sounds. Since the most sensitive neurons determine neuronal and behavioral thresholds, hearing loss is expected to produce elevated thresholds (Slade et al., 2020), as has been shown for example in auditory brainstem responses that are elevated by ∼30 dB comparing young (6 months) to older (12 months) rats (Alvarado et al., 2012, 2014).

Our neural classifier results are similar to related decoding work in other auditory brain areas based on spiking activity. In one study, 96-channel intracortical array spiking activity recording in macaque auditory cortex was used for a five-class classification, investigating a large number of different classifiers. Optimal classifier performance of >0.9 was achieved with performance reaching asymptotic values at ∼50 channels (Heelan et al., 2019). Likewise, another study used unit recordings from rat primary auditory cortex to classify responses to 18 PTs and achieved performance of up to ∼50% accuracy at a chance level 5.5% (Szabó and Barthó, 2022). This performance is somewhat lower than the 90% we observed at some frequencies, which could be due to various factors including the number of classes, recording channels, or precise choice of algorithm. In addition to these decoding studies on spiking activity, decoding analyses have also been performed in humans based on noninvasive signals, obtained, for example, using near-infrared spectroscopy or functional magnetic resonance imaging (Zhang et al., 2015; Yoo et al., 2021). In these cases, decoding performance tends to be substantially lower than for spiking activity, e.g., reaching performance values of ∼30% for a seven-class classification with a chance level of 14%, highlighting both the advantage of invasive recordings for fine-grained stimulus classification and the feasibility of simultaneously decoding multiple frequency classes using supervised algorithms. Overall, the large channel count of modern multichannel probes now permits excellent decoding performance of auditory stimuli from auditory pathway structures including the thalamus. This enables precise assessment of auditory information encoding at the single-subject level, which opens new possibilities for medical device development (Shaeri et al., 2024) as well as diagnosis and preclinical treatment assessments of auditory disorders.

## Synthesis

Reviewing Editor: Anne Keitel, University of Dundee Decisions are customarily a result of the Reviewing Editor and the peer reviewers coming together and discussing their recommendations until a consensus is reached. When revisions are invited, a fact-based synthesis statement explaining their decision and outlining what is needed to prepare a revision will be listed below. The following reviewer(s) agreed to reveal their identity: Yoshinao Kajikawa, Bernhard Gaese.

The manuscript has been assessed by two expert reviewers and the editor. Both reviewers find the manuscript generally well-written with interesting results but raise significant methodological concerns about comparing behavioural data from awake animals to neural recordings under anaesthesia, and critically note missing details about the acoustic parameters of the narrow band noise stimuli. The reviewers question why MGN rather than cortex was studied given the behavioural focus, and request clarification on numerous technical points including electrode configuration, statistical methods, and potential confounds from stimulus envelope fluctuations. Based on these and other points (see below) we have come to the conclusion that the manuscript can only be considered for publication after a major revision.

You will find a synthesis of both reviewers' comments below. Please respond to each comment in a point-by-point manner.

Overall assessments for your information

(1) This study investigated the properties of MGN responses to pure tone (PT) and narrow band noise (NBN) sounds in rats, with the possible development of rodent model for preclinical evaluations in mind. To compare with neuronal data, the study also examined performance of behavioral task as well. They show that threshold for detection of PT and NBN sounds are similar between behavior and MGN neurons. They show further for those neurons that, while PT and NBN responses have similar spectral tuning, they differ in several response properties. Lastly they addressed neurons' ability to discriminate frequency bands of sounds. The manuscript appears written well. However, I have several concerns.

(2) The ms. provides data from behavioral testing of rats in an auditory detection task of pure tone and narrow-band noise stimuli depending on sound pressure level. Comparable neuronal data are provided from unit recordings in the medial geniculate body under anesthesia. Sophisticated analysis was applied to quantify detection performance in both data sets, trying to determine the difference in performance depending on stimulus types.

Behavioral testing with the additional factor of stimulus onset delay in different test trials provides very good basis to determine the mentioned discrimination performance. The neuronal recordings provide a good overview of frequency encoding and SPL-dependence of activity in the auditory MGB. The results at the unit level mainly confirm nicely what previous studies had also revealed. The sophisticated detection analysis adds some interesting aspects, especially regarding the difference in parameter encoding between PT and NBN. The ms, however, describes a lot of effects without providing insight into possible mechanisms.

The analysis at the population level also reveals interesting results. These, however, cannot be fully appreciated without knowing all the relevant stimulus parameters of NBN stimuli (see below).

The critical point in this ms is the comparison between narrow-band noise (NBN) and pure tones (PT). Both stimulus types generate different levels of neuronal excitation and behavioral performance. The validity and robustness of these differences can only be estimated if additional information about measurement procedures and acoustic parameters of these stimuli are added to the ms.

Major comments - please respond to these

Conceptual/discussion points

1. While the behavior was assessed in awake animals, neurons were assessed under anesthesia. Influence of anesthesia on neurons (e.g., sensitivity, tuning, other response parameters, etc.) should be mentioned or discussed.

2. While this study examined MGN responses, behavior is often linked to cortex. Also, the first paragraph in Introduction describes scalp signal responses of human subjects. Those usually originate from cortex as well. Page 15, "To examine the neural underpinnings of these behavioural results," Structure underpinning behavior would not be cortex? The manuscript does not appear to describe well why they studied MGN.

3. Authors should mention that only single band was tested in behavioral task. It is not clear if sound detection in other bands would be similar. It would be nice to discuss the possibility of the behavioral performance extend to other frequency bands.

4. Discussions comparing human studies and results in the manuscript appear not comparing the bandwidth of sounds used in studies. E.g., Is the spectral spread for tinnitus activation comparable to 7-10kHz NBN?

5. Page 22, "Slower response dynamics for visual pathway complex cells and auditory neurons responding to NBN stimuli may thus reflect additional recurrent processing that occurs in these two cases, reflecting common processing across sensory modalities" This argument appears misleading. While simple and complex cells are different neurons exhibiting different responses, same cells responded differently to PT and NBN in this study.

6. Page23, authors attribute the possible mechanism of "The reversal in preference" to MGBv and MGBd. But it is possible that processing of PT and NBN in primates is different from rodents.

7. Page 23 "However, we suggest that they support auditory processing in the intermediate and high decibel range, where units with low C50 are no longer sensitive due to the broadening of their tuning function. This could explain why hearing loss tends to produce elevated thresholds" I am not sure of logic here, why the description in the first sentence leads to that of the second sentence.

Methods

8. As seen for example in Fig. 4 in alle FRAs the most sensitive neurons all had minimal thresholds above 35 dB SPL. This is different from most recordings in MGB under anesthesia, very often also neurons down to 20 or even 10 dB SPL have been found. This depends (among other factors) on the type and depth of anesthesia and on the background noise level. Any indication of what the limiting factors are in this study?

9. page. 5 / lines 1-3:

explain better how the stimulation arrangement was in the behavioral task. A speaker "50 cm above the chamber", earlier defines as "sound attenuated operant chamber" seems not appropriate.

What sound level had the acoustic background in the chamber? Was this comparable between behavioral tests and electrophysiological recording?

10. p 5 / l 18:

What was the bandwidth of NBN stimuli?

11. p 7 / l 1:

Are frequencies given for the total range or NBN center frequencies? Again, what was the NBN bandwidth?

12. Related to points 3 and 4: NBN for task is 7-10kHz which is 0.515 octave wide. I presume that the bandwidth for the task is set on linear frequency scale. What is the bandwidth of NBN for e-phys? Those are spaced on logarithmic scale.

13. How are electrode channels configured in this study?

Are analyzed units, particularly for decoding, from simultaneous recording? While the last paragraph in Discussion describes other studies of decoding, aren't those studies decode simultaneously recorded signals?

14. Page 12, "10 pseudo-populations were generated" The description is vague about how those populations were generated. i.e., 10 times totally random selection with or without overlap of units between populations, uniform selection of units that have different CF,C50, etc.

15. Methods should describe what statistics and criterion levels are use.

16. Page 9, in log-normal function, baseline firing rate "B" is described as one of free parameters. Isn't it based on actual spontaneous firing rate?

I think the fitting curves in Fig 2 fit data points nicely. However, the parameter B could vary unreasonably across tone frequencies, depending on and correlating with C50. Particularly for frequencies of low C50, B appears to take negative values.

17. Page 11, upper bound of time constant is described as 1. Is it correct? Fig.5F shows longer time constants.

18. Page 12, in expression of Pc, "D=0" may need more description to explain it, because it is unlikely to see often exactly D=0. I speculate that the cases of D<0 are counted as D=0.

Results

19. p 15 / l 4: Statistical evidence for the observed anti-correlation is derived from t-tests. Please explain.

20. p 15 / l 12-15: Not many papers do acute recording under isoflurane anesthesia. It should be noted clearly at this point if the data were recorded under ketamin/xylazine (initial phase) or isoflurane influence (after initiation).

21. p 16 / l 14: Regarding the tonotopy it should be noted in the text that only the lower part of the rat audiogram was tested in this ms for representation.

22. p 18 / ls 10-16 /Fig. 5F-H: The differences in response timing between stimulus types strongly depends on the envelope waveform of stimuli. In the case of NBN stimuli, the narrower the band is, the more fluctuating is the stimulus envelope. This might explain the differences found, including a less sharp stimulus onset which determines the height of the initial response peak. Any indication here that this is not resulting in the differences found?

23. Fig 1:

(C) It should be explained more clearly what was averaged for each point in the plot. As it looks like about 8 points per color/SPL one can assume that each plot includes data from one animal across all test sessions

(D) Same as for (C)

(E) State clearly what data are included in one data point (presumably all data for a given animal at one SPL)

24. Fig. 2:

(A) (A) The C50-point in the FRA shown is here (as for the other FRAs) rather close to the threshold between response area and background/spontaneous activity. One might assume that this results from max. response activities being rather low. Please provide the distribution of max response activities found compared to the level of neuronal background activity. Different from the normalized rates, thereby one can get a feeling for the signal/noise rations in these neurons.

(B) If data shown here are from the same unit as in Fig. 2A (as noted), why is there a difference in CF (in A: 3.2 kHz, in B: 2.2 kHz)? The resulting C50s also do not match. Check your data for consistency!

25. Fig. 1B legend, "same animal as in (B)" should be "the same animal as in (A)"

26. Fig. 2D legend, the last sentence about definition of o-shape is vague. Is criterion more objective?

27. Fig. 3C legend needs explanation of what asterisks are.

28. Figure legends may not need asterisks in texts where no asterisks are present in Figure itself.

29. Fig.4 and 5. I realize that Fig4 includes all data including ones that had responses to only PT or NBN and Fig5 uses subset that had responses to both. It would be appreciated if authors explicitly state that Fig4 includes all data in main texts.

30. Fig.5 shows shorter latency and higher peak magnitude of responses to PT than NBN, even though overall firing rate is similar. It is described that "in response to the amplitude eliciting highest activity at the CF" is analyzed. As C50 is lower for NBN than PT, such best SPL may not be same between two sounds. It would be nice if authors add description about that. Response latencies and ISI may correlate with sound levels. I suggest examining such relationships and compare between PT and NBN responses as well.

Also, Fig.5F-H panels suggest that the duration of responses is longer for NBN. Is that correct? Is it possible that onset responses to pure tone are followed by inhibition while the onset responses to NBN are not?

31. What is Fig.7E is not clear. Even though written as cross validation, is it actually self validation? Should "(A)" in "the drop in (A)" be "(D)?"

32. How many tracks per animal? The number of slices in Fig.3-1 does not match the number of animals.

Extended Data

33. Fig 1-1: Wrong labeling on X-axis

Minor Points - please respond to these

1. Always include a space (blank) between the unit of measurement and the number

p 16 / l 4 from bottom: should be: "Example FRAs from single units illustrate the ..."

2. Page 8, in the equation of behavioral sensitivity, d-prime, "Z(HR)" probably should be "Z(HT)."

3. Page 12, typo, "whre"

4. Page19,"p<<0.001." Probably just one inequality is enough.
