# Supplementary figures and images for: Neural and Behavioral Correlates of Pure Tone and Narrowband Noise Processing in Rats: A Tradeoff between Discrimination and Sensitivity
Source: eNeuro. 2026 Apr 8;13(4):ENEURO.0347-25.2026. doi: 10.1523/ENEURO.0347-25.2026 (PMC13075772; doi:10.1523/ENEURO.0347-25.2026)

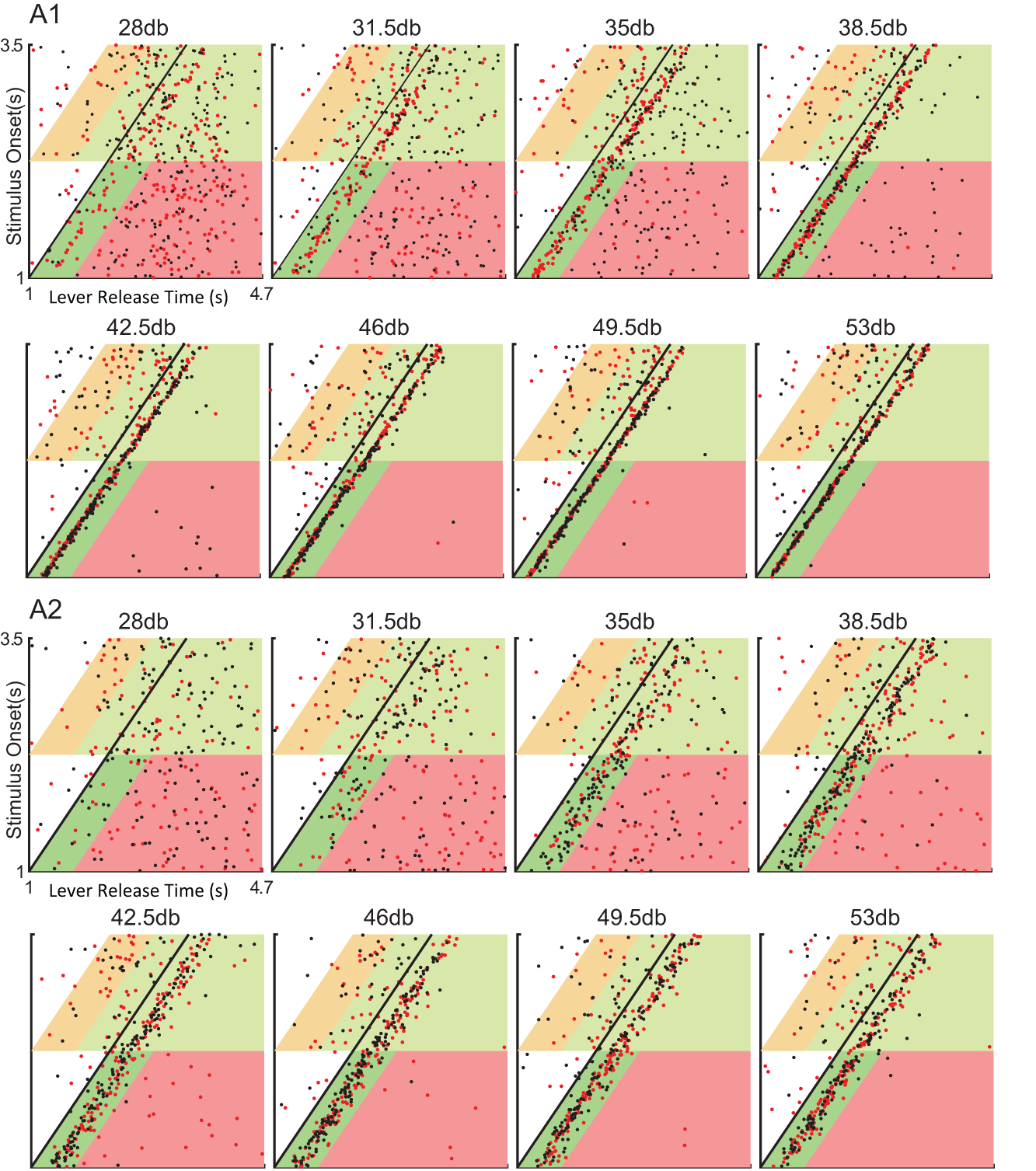

Supplement: Figure 1-1 — By displaying the holding time of each animal at each amplitude, it is possible to observe the distinct strategies developed and adopted by each individual. Nonetheless, all animals showed a similar overall trend: a decrease in correct responses and an increase in errors as the stimulation amplitude was lowered. Download Figure 1-1, TIF file. [file eneuro-13-ENEURO.0347-25.2026-s003.tif]

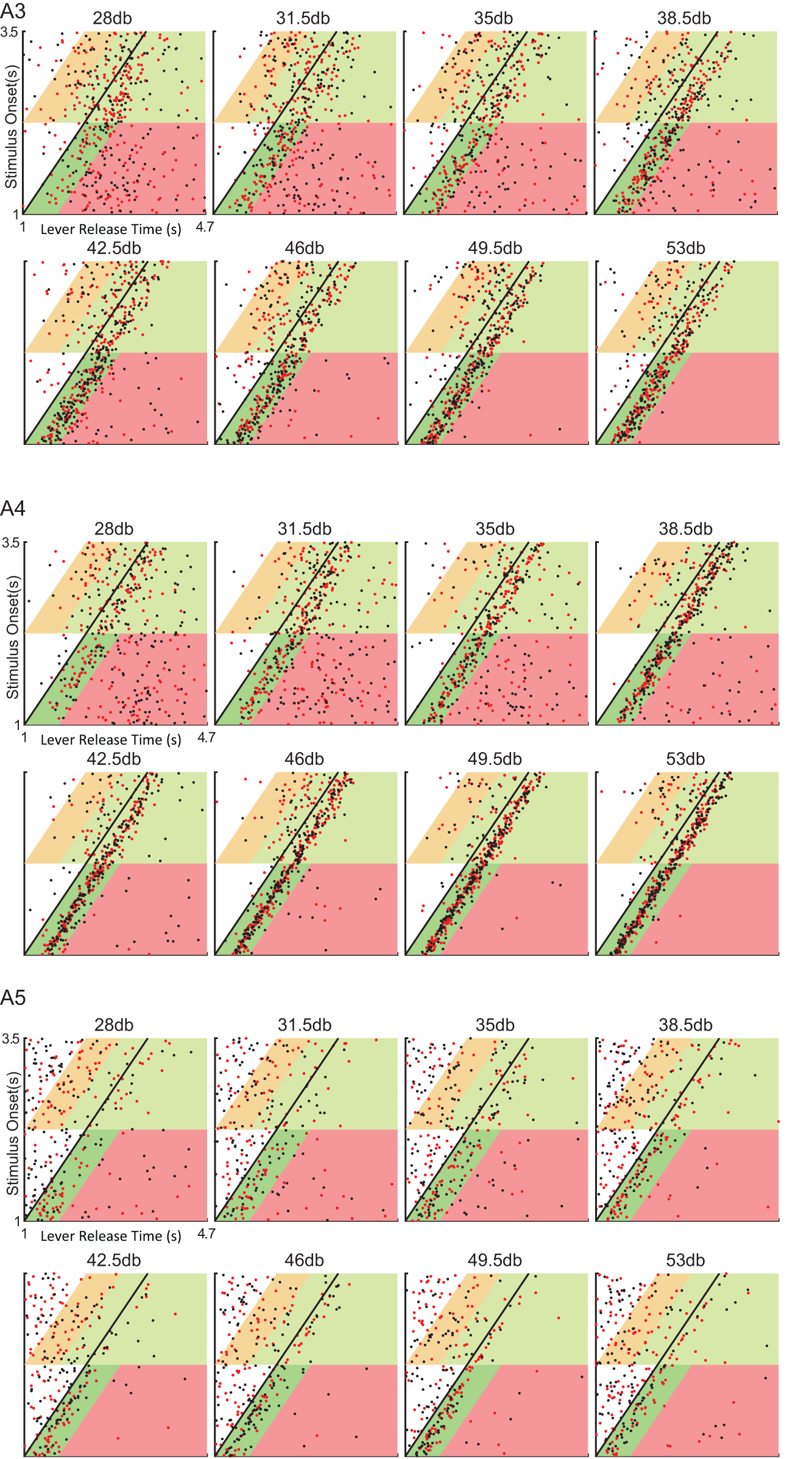

Supplement: Figure 1-2 — By displaying the holding time of each animal at each amplitude, it is possible to observe the distinct strategies developed and adopted by each individual. Nonetheless, all animals showed a similar overall trend: a decrease in correct responses and an increase in errors as the stimulation amplitude was lowered. Download Figure 1-2, TIF file. [file eneuro-13-ENEURO.0347-25.2026-s004.tif]

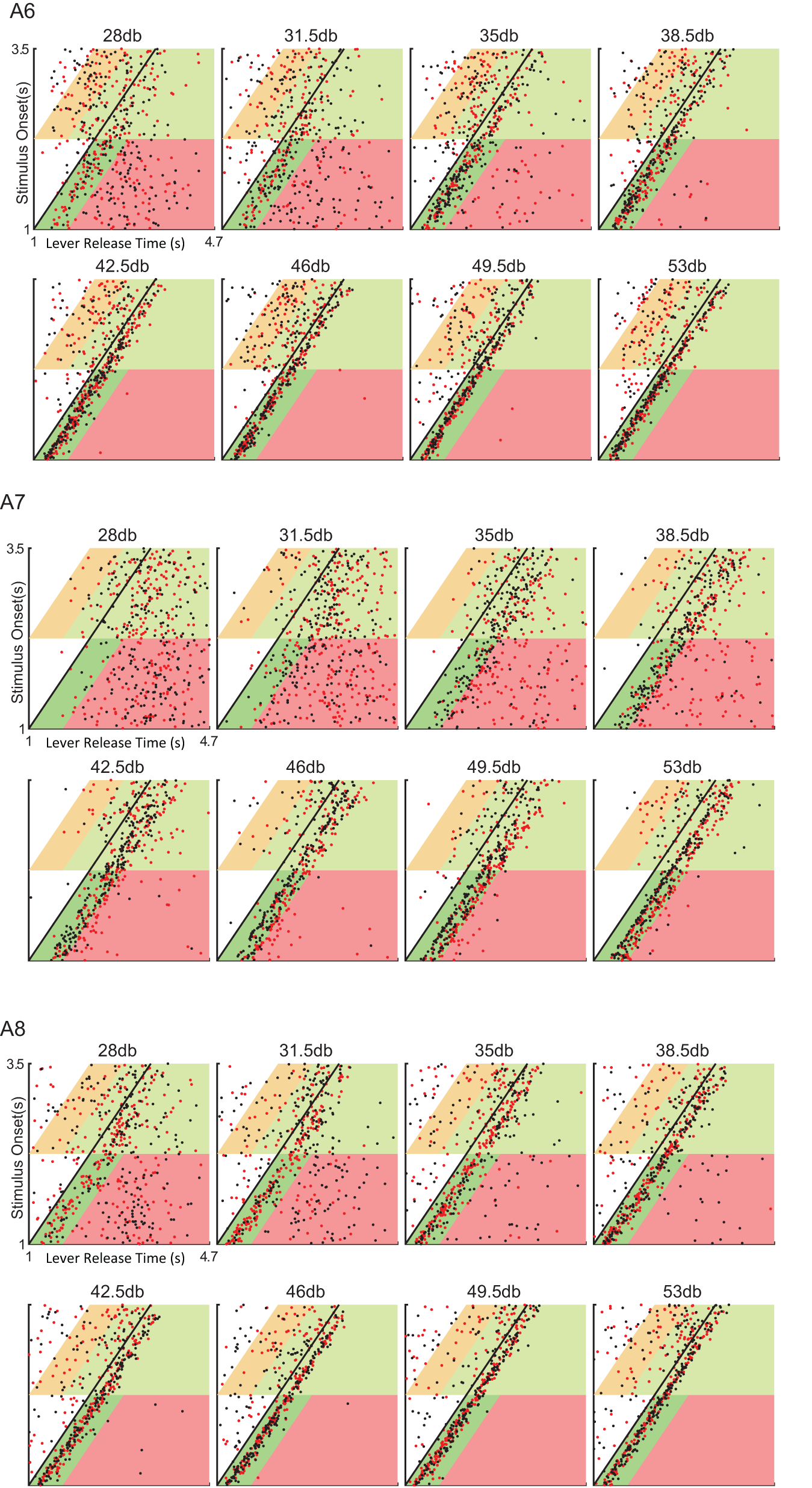

Supplement: Figure 1-3 — | Single animal behavioral performances A6-A8. Lever release times for all individual animals (A6-A8) at all amplitudes (28; 31.5; 35; 38.5; 42.5; 46; 49.5; 53 dB SPL). The black oblique line marks the target onset. Colored trapezoids indicate different trial outcomes: dark-green hit (H), red miss (M), yellow false alarm (FA), light green correct rejection (CR), and white abort (AB). Download Figure 1-3, TIF file. [file eneuro-13-ENEURO.0347-25.2026-s002.tif]

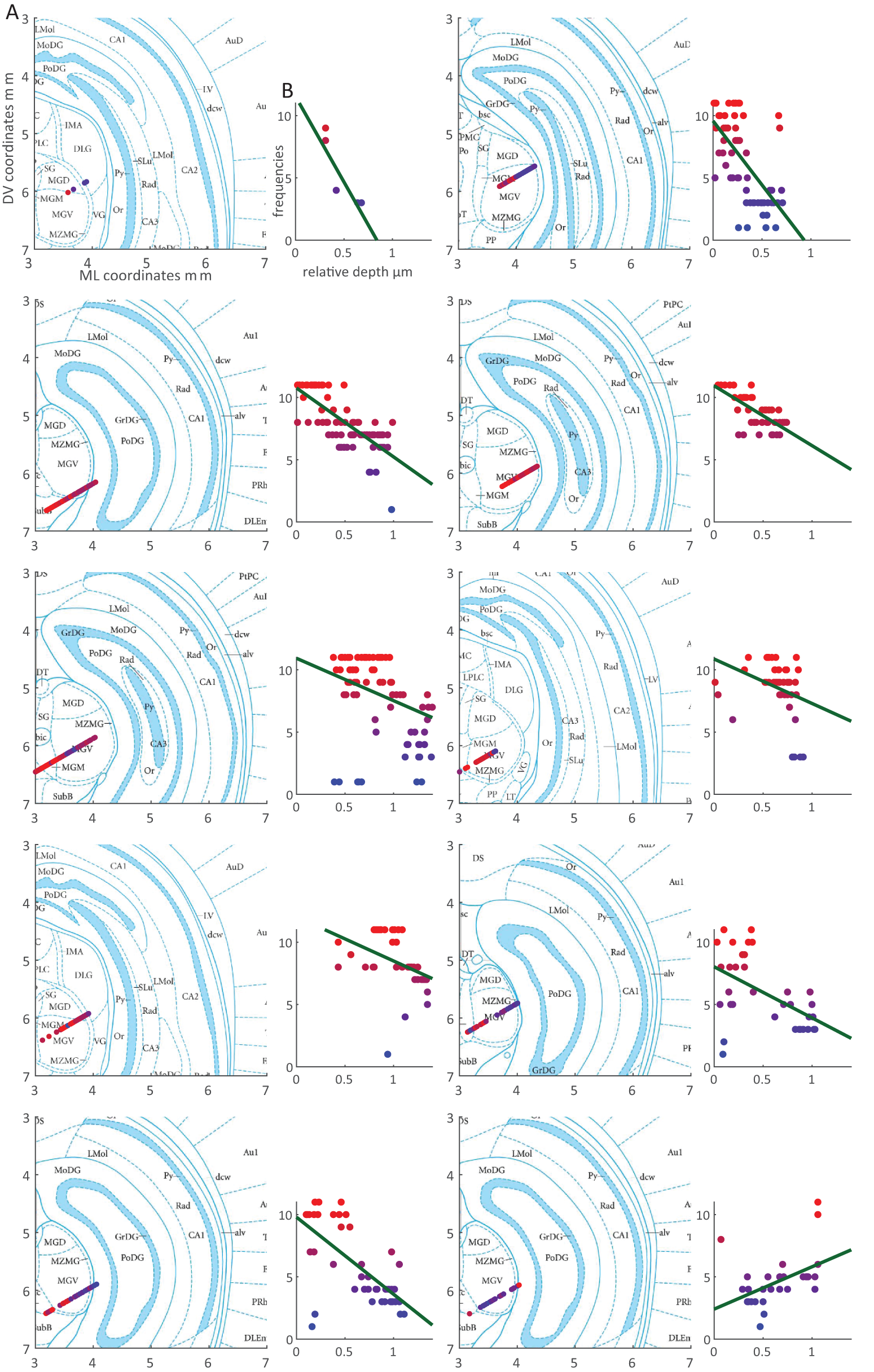

Supplement: Figure 3-1 — | Tonotopic organization of the rat auditory thalamus (MGB). A. Schematic coronal representation of the rat MGB showing the relative unit positions, color coded by their CFs, for all the penetration with a significant recorded response; the scale goes from blue, low frequency, to red, high frequency. B. A scatterplot showing CF against distance from the tip of the probe for the recorded units, with a fitted linear regression line. Download Figure 3-1, TIF file. [file eneuro-13-ENEURO.0347-25.2026-s005.tif]

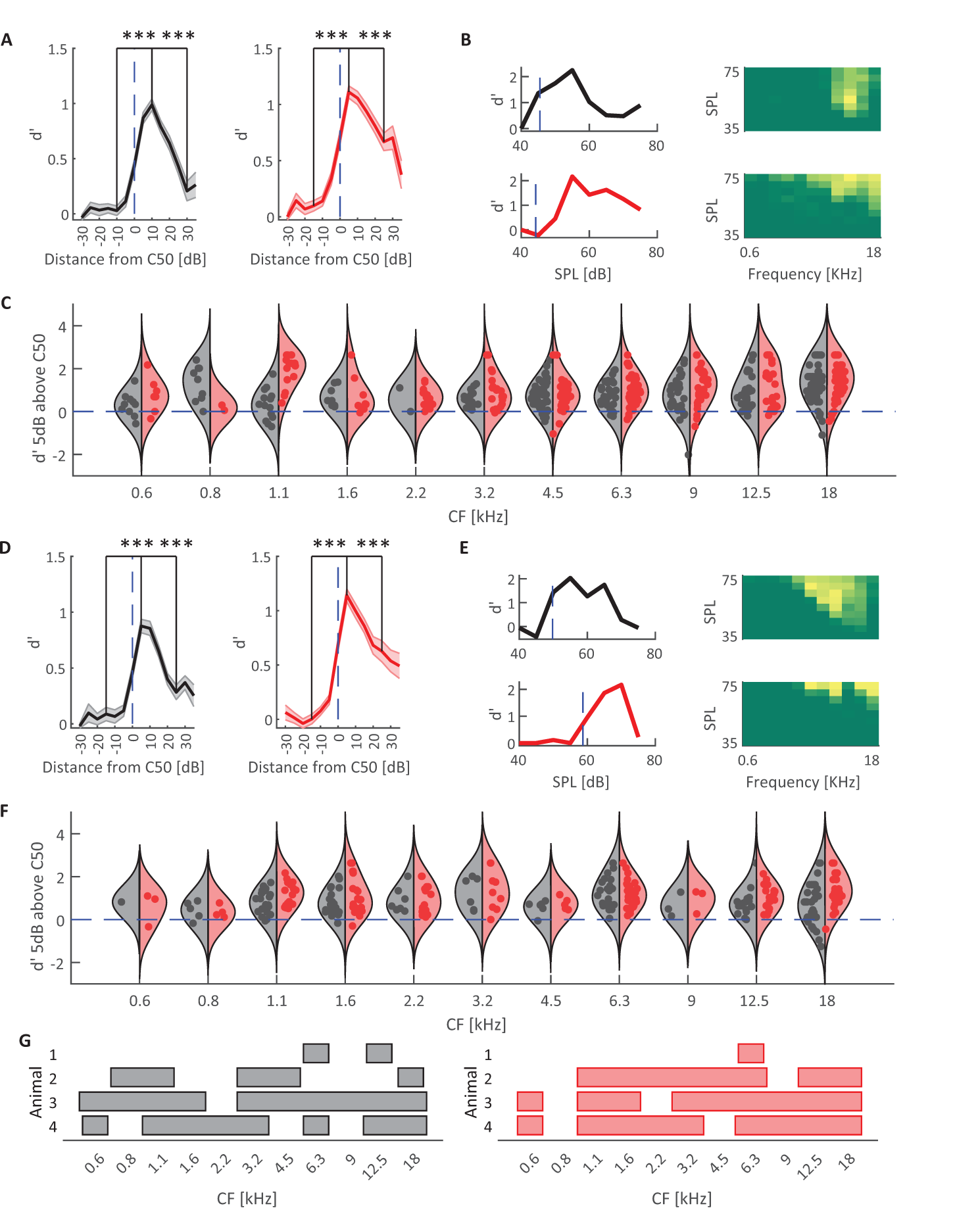

Supplement: Figure 6-1 — | Discriminability (d’) of narrowband noise (NBN) and pure tone (PT) single animals. A-D. Mean d’ values as a function of distance from each unit’s C50 for 2 example animal (animal 3 – A, animal 4 - D). NBN (left, black) and PT (right, red) both show a sharp drop in d’ for stimuli ± 20 dB from d’ peak situate 5-10 dB above C50 (p < 0.001, paired t-tests). Shaded areas represent SEM. B-E. Examples coming from 2 different subjects (animal 3 – B, animal 4 - E) of d’ values across amplitudes for two individual units responsive to NBN (top) and PT (bottom), blue dashed lines indicate unit’s C50. Corresponding FRA are shown on the right. C-F. Violin plots showing the distribution of d’ values computed 5 dB above C50 across sound levels for units responsive to both NBN (black) and PT (red), plotted as a function of characteristic frequency (CF) for 2 example animal (animal 3 – C, animal 4 – F). Each violin represents a frequency band, with individual unit data overlaid. A significant main effect of both frequency and stimulus type was found for animal 3 while we only found a main effect of frequency for animal 4 (two-way ANOVA, C: p < 0.001 – frequency and p < 0.05 – stimulus type; F: p < 0.05 – frequency and p > 0.1 – stimulus type). G. Summary figure showing for each subject in which frequency bands the average peak d’ exceeded 1 across the recorded units for NBN (left) and PT (right). Download Figure 6-1, TIF file. [file eneuro-13-ENEURO.0347-25.2026-s006.tif]

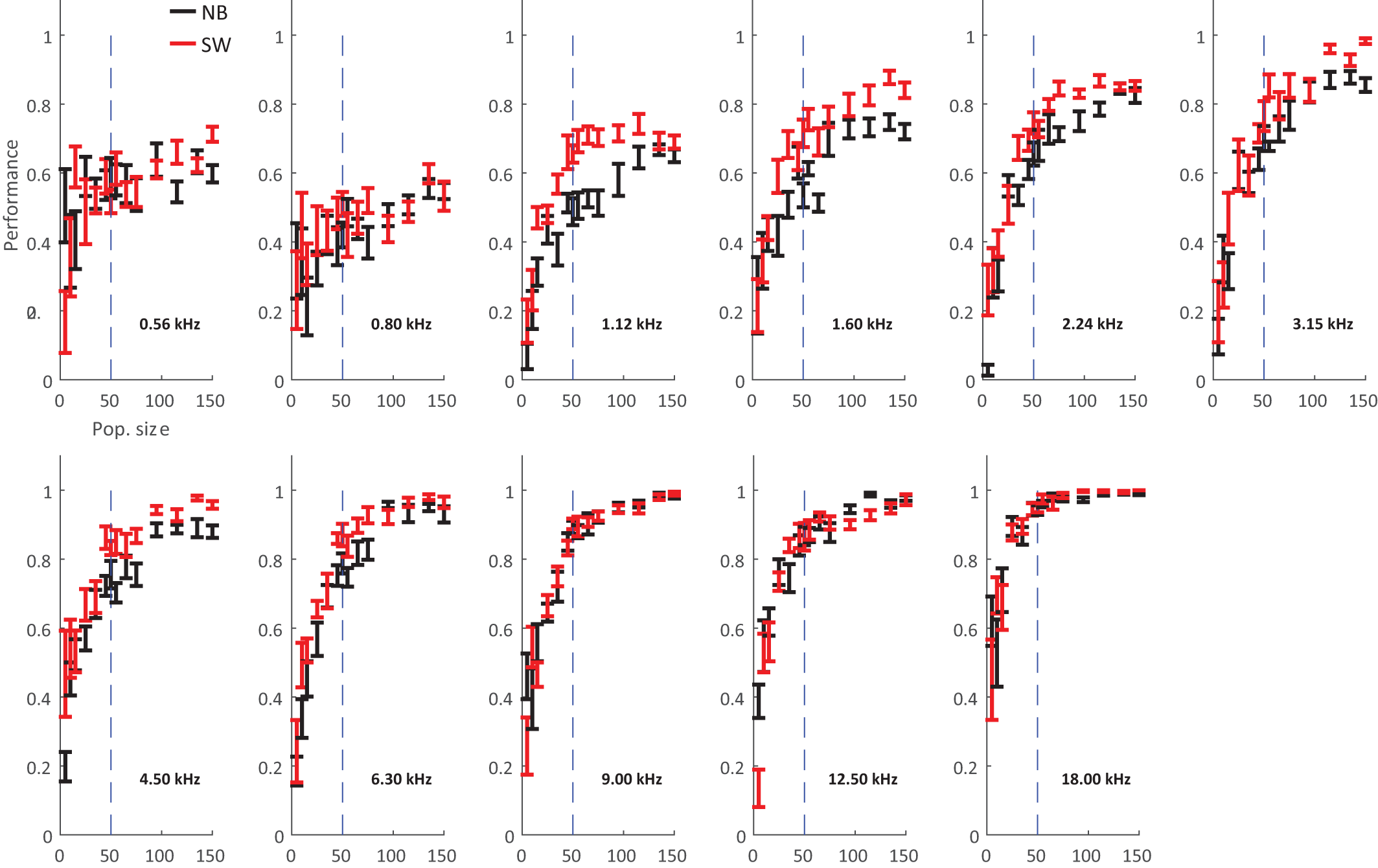

Supplement: Figure 7-1 — | Decoding performance as a function of population size across frequencies. Decoding accuracy is shown for increasing numbers of units at different center frequencies, comparing NBN (black) and PT (red) stimuli using the 0–50 ms response window. Error bars denote ± SEM across 10 random populations. The vertical dashed blue line marks a population size of 50 units, which was used for all main decoding analyses. At most frequencies, performance improved steeply with population size up to ∼50 units, after which gains plateaued and additional units contributed little to further improvement. Download Figure 7-1, TIF file. [file eneuro-13-ENEURO.0347-25.2026-s007.tif]

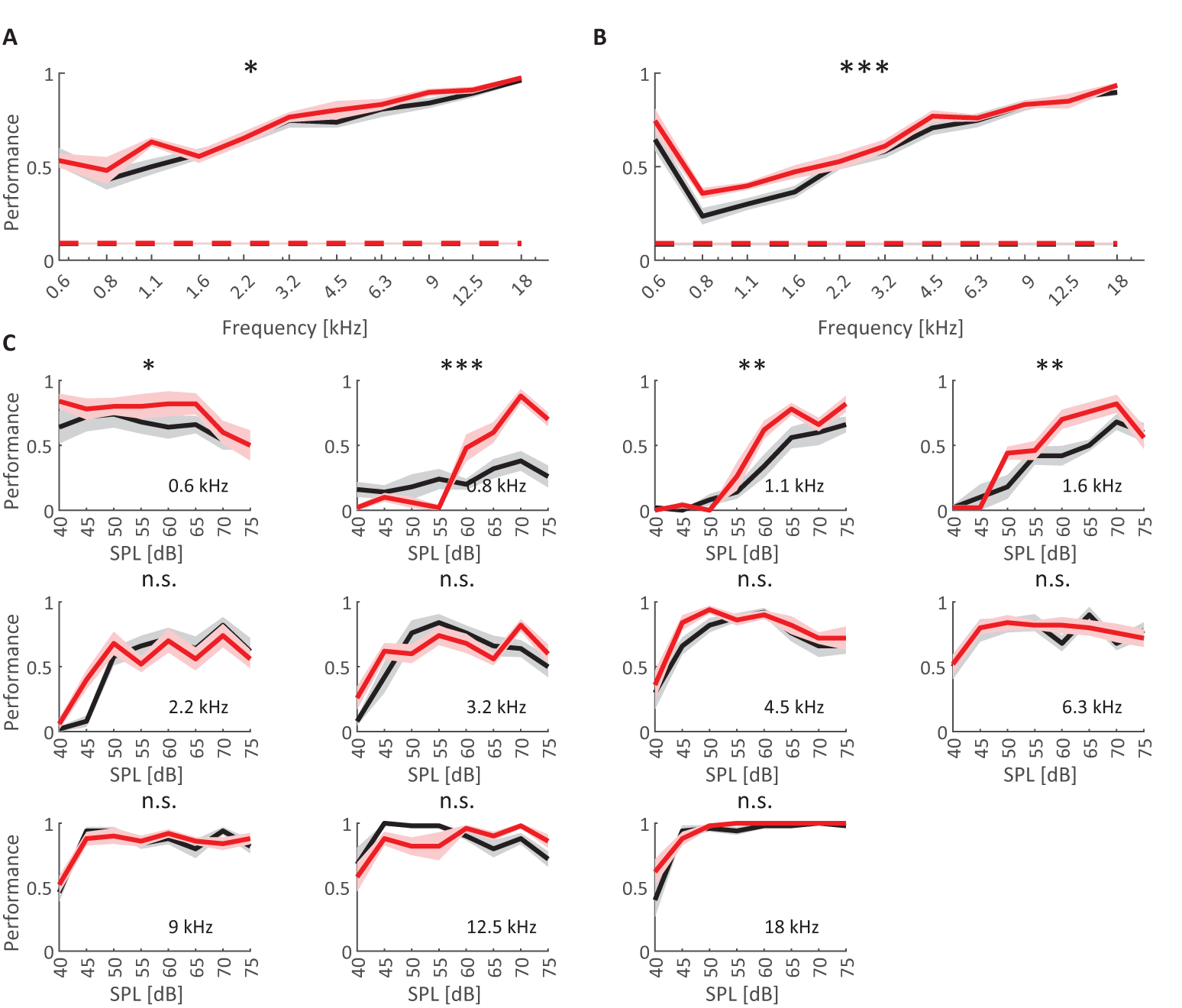

Supplement: Figure 7-2 — | Decoding performance for frequency discrimination animal 3. A-B. Multiclass SVM decoding performance for predicting NBN and PT stimulus frequency based on spike count responses from populations of 50 neurons (average across 10 populations for each condition). Performance has been evaluated for two temporal windows: the longer response window (0-50 ms, A) used in d’ analysis, and an early response window (0-25 ms, B) to investigate the inpact of the differences in response timing. A. During the 50 ms response window, decoding accuracy was significantly different between PT and NBN, with PT achieving higher performances (p < 0.05, two-way ANOVA). We also observed a significant increase of decoding accuracy with frequency (p < 0.001, two-way ANOVA). B. In the early response window (0-25 ms), decoding performances were even higher for PT (red) compared to NBN (black) stimuli across frequencies (p < 0.001, two-way ANOVA). Shaded areas represent ± SEM across the 10 populations. Horizontal dashed line indicates chance level (∼9.1%, based on shuffled labels). C. Decoding performance as a function of sound pressure level (SPL) for a given center frequency, comparing NBN and pure tone PT stimuli using the early response window as in B. A two-way ANOVA revealed a significant effect of stimulus type at 560 Hz (p < 0.05), 800 Hz (p < 0.001), 1120 Hz (p < 0.01), 1600 Hz (p < 0.01), with PT stimuli consistently yielding higher decoding accuracy than NBN. No significant effect of the stimulus type was observed at other frequencies. Download Figure 7-2, TIF file. [file eneuro-13-ENEURO.0347-25.2026-s008.tif]

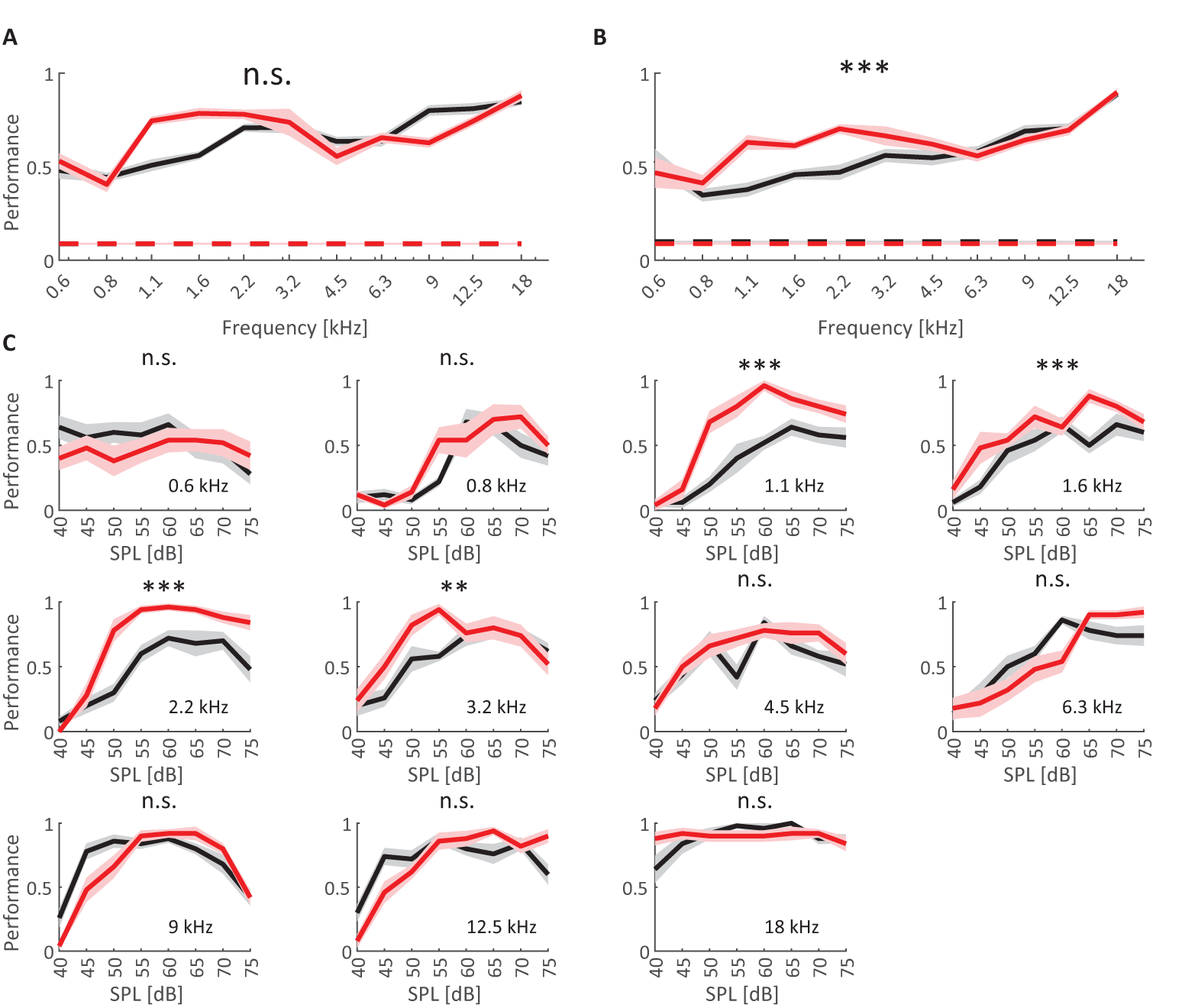

Supplement: Figure 7-3 — | Decoding performance for frequency discrimination animal 4. A-B. Multiclass SVM decoding performance for predicting NBN and PT stimulus frequency based on spike count responses from populations of 50 neurons (average across 10 populations for each condition). Performance has been evaluated for two temporal windows: the longer response window (0-50 ms, A) used in d’ analysis, and an early response window (0-25 ms, B) to investigate the inpact of the differences in response timing. A. During the 50 ms response window, decoding accuracy was not significantly different between PT and NBN (p = 0.053, two-way ANOVA). We observed a significant increase of decoding accuracy with frequency (p < 0.001, two-way ANOVA). B. In the early response window (0-25 ms), decoding performances were higher for PT (red) compared to NBN (black) stimuli across frequencies (p < 0.001, two-way ANOVA). Shaded areas represent ± SEM across the 10 populations. Horizontal dashed line indicates chance level (∼9.1%, based on shuffled labels). C. Decoding performance as a function of sound pressure level (SPL) for a given center frequency, comparing NBN and pure tone PT stimuli using the early response window as in B. A two-way ANOVA revealed a significant effect of stimulus type at 1120 Hz (p < 0.01), 1600 Hz (p < 0.01), 2240 Hz (p < 0.001), 3150 Hz (p < 0.01), with PT stimuli consistently yielding higher decoding accuracy than NBN. No significant effect of the stimulus type was observed at other frequencies. Download Figure 7-3, TIF file. [file eneuro-13-ENEURO.0347-25.2026-s009.tif]
